# Supplementary material for: Graves-PCD: protocol for a randomised, dose-finding, adaptive trial of the plasma cell-depleting agent daratumumab in severe Graves’ disease
Source: BMJ Open. 2024 Jun 12;14(6):e079158. doi: 10.1136/bmjopen-2023-079158 (PMC11177693; doi:10.1136/bmjopen-2023-079158)
Supplement: Supplementary data [file bmjopen-2023-079158supp001.pdf]

```
library(boot)
library(MCPMod)
library(DoseFinding)


#Assume that -log(percentage of baseline) (-log of ratio of follow-up TRAb to baseline TRAb) is normally distributed and the outcome


#Function to run a given number of simulation replicates:

simulation=function(
  initialdoses=c(0,0.5,1,3,9), #Initial range of doses given
  stage1.nperdose=3, #Initial number of patients per dose in stage 1
  maximumpercentagereduction=80, #plateau percentage reduction from baseline
  minimumpercentagereduction=5,#mean reduction with placebo
  ed50=2,#dose that gives 50% of effect
  errSD=sqrt(1/4),#SD of transformed outcome (assuming the SD is 25 at the mean of 50% reduction) is approximately sqrt(1/50^2 * 25^2)
  alpha=0.05, # the alpha level
nreplicates=1000 #number of simulation replicates to run
)
{
  #define vectors to track statistical properties

  power.contrast=rep(0,nreplicates)
estimated.dose50=rep(NA,nreplicates)
estimated.ed50=rep(NA,nreplicates)

selectedstage2doses=matrix(NA,nreplicates,3)
xdose<-initialdoses
nsam<-stage1.nperdose #number of patient per dose

#Hypothesised dose response

#transform of maximum %age reduction
ehi<- -log((100-maximumpercentagereduction)/100)
#Transform for placebo reduction
elo<- -log((100-minimumpercentagereduction)/100)


for(replicate in 1:nreplicates)
{

#sim first stage data
dfData.stage1 <- genDFdata("emax", c(e0 = elo, eMax = ehi-elo, ed50 = ed50), doses=xdose, sigma=errSD, n=nsam)


#plot(dfData.stage1$dose,dfData.stage1$resp)
#points(aggregate(dfData.stage1$resp, list(dose=dfData.stage1$dose), mean),pch=15)

#fit an ANOVA model to estimate means
fit.aovmean = lm(resp~as.factor(dose)-1, data=dfData.stage1) #no intercept
#summary(fit.aovmean)
EstMean.aov = coef(fit.aovmean) # Extract the estimated sample means
aov.sigma = summary(fit.aovmean)$sigma # Extract the estimated sigma

# PoC: contrast mean >0 statistically?
len.Dosage<-length(xdose)

n=nsam # sample size for each dosage
# The c's for 4-dose and 1-placebo
Contrast.Coef = c(-2,-1,0,1,2)
# Implementation of equation 9.2. with 1-sided test "Dose Finding in Drug Development" Naitee Ting. Springer
# where "qt" is for the quantile-t with one-sided (i.e. 1-alpha)

teststatistic.contrast=sum(Contrast.Coef*EstMean.aov)/(aov.sigma*sqrt(sum(Contrast.Coef^2/n)))
pvalue=1-pt(teststatistic.contrast,df=(n-1)*len.Dosage)
PoC.Contrast=sum(Contrast.Coef*EstMean.aov)/(aov.sigma*sqrt(sum(Contrast.Coef^2/n))) >qt(1-alpha,df=(n-1)*len.Dosage)

# fit the "Emax" model
fitemax = fitMod(dose, resp, data=dfData.stage1, model="emax",  bnds = c(0.01, 25))
# Call "plot" to plot the Emax fit
#plot(fitemax,CI=TRUE,plotData="meansCI",xlab="Dose",ylab="Response")


#function to find dose achieving reponse of 50% and bootstrap CI



#If e0+eMax is less than the equivalent of a 25% reduction, then stop early:

if((fitemax$coefs[1]+fitemax$coefs[2])< (-log(0.75)))
{
  next;
}

#If e0+eMax is between 25% and 50% reduction, then second stage doses should be 0, 3 and 9:

if((fitemax$coefs[1]+fitemax$coefs[2])< (-log(0.5)))
{

  xdose.stage2.overrun<-c(0,0.5,1,3,9)
  dfData.stage2.overrun <- genDFdata("emax", c(e0 = elo, eMax = ehi, ed50 = ed50), doses=xdose.stage2.overrun, sigma=errSD, n=1)
  xdose.stage2.afterinterim=c(0,3,9)

  selectedstage2doses[replicate,]=c(0,3,9)
```

```
}

#If e0+eMax is higher than 50% reduction, then select dose that gives 50% reduction and one that gives 90% of plateau reduction
if((fitemax$coefs[1]+fitemax$coefs[2])>= (-log(0.5)))
{

#extra dose chosen to be close to plateau effect:
plateau=fitemax$coefs[1]+fitemax$coefs[2]

#find dose that gives 90% of plateau effect:

dose.nearplateau=doseforgivenmean(fitemax,mu=0.9*plateau)

dose.nearplateau=round(dose.nearplateau)



#Estimated dose that gives 50% reduction, or 50% of plateau if plateau is lower than 50:

dose.50=doseforgivenmean(fitemax,mu=-log(0.5))
#round to nearest dose:

dose.50=round(dose.50)
#If rounded dose.50 is less than or equal to 0 (can be less if e0 is estimated to be high), set to 0.5:

if(dose.50<=0)
{
  dose.50=0.5
}

#If rounded dose.50 is >9, set to 6:

if(dose.50>9)
{
  dose.50=6
}

#If rounded dose.plateau is <= dose.50, set to dose.50+1:

if(dose.nearplateau<=dose.50)
{
  dose.nearplateau=dose.50+1
}

#If rounded dose.nearplateau is >9, set to 9:

if(dose.nearplateau>9)
{
  dose.nearplateau=9
}



#To allow for overrun, secondstage doses include 5 more recruited under the stage 1 allocation, then remaining allocated 3,3,3 to new
#Note this only makes 29

xdose.stage2.overrun<-c(0,0.5,1,3,9)
dfData.stage2.overrun <- genDFdata("emax", c(e0 = elo, eMax = ehi, ed50 = ed50), doses=xdose.stage2.overrun, sigma=errSD, n=1)
xdose.stage2.afterinterim<-c(0,dose.50,dose.nearplateau)

selectedstage2doses[replicate,]=c(0,dose.50,dose.nearplateau)

}

dfData.stage2.afterinterim <- genDFdata("emax", c(e0 = elo, eMax = ehi, ed50 = ed50), doses=xdose.stage2.afterinterim, sigma=errSD, n=

dfData.combined=rbind(dfData.stage1,dfData.stage2.overrun,dfData.stage2.afterinterim)


#Final analysis:

#fit an ANOVA model to estimate means
fit.aovmean = lm(resp~as.factor(dose)-1, data=dfData.combined) #no intercept
summary(fit.aovmean)
EstMean.aov = coef(fit.aovmean) # Extract the estimated sample means
aov.sigma = summary(fit.aovmean)$sigma # Extract the estimated sigma

#Set up contrast: check if even or odd number of unique doses

ndoses=length(unique(dfData.combined$dose))
if(ndoses==5){Contrast.Coef<-c(-2,-1,0,1,2)}
if(ndoses==6){Contrast.Coef<-c(-5,-3,1,1,3,5)} #notice change in contrast from -3,2,-1,1,2,3 so equal spacing throughout
if(ndoses==7){Contrast.Coef<-c(-3,-2,-1,0,1,2,3)}



n=as.double(table(sort(dfData.combined$dose))) # sample size for each dosage

# Implementation of equation 9.2. with 1-sided test "Dose Finding in Drug Development" Naitee Ting. Springer
# where "qt" is for the quantile-t with one-sided (i.e. 1-alpha)


teststatistic.contrast=sum(Contrast.Coef*EstMean.aov)/(aov.sigma*sqrt(sum(Contrast.Coef^2/n)))
```

```
pvalue=1-pt(teststatistic.contrast,df=2*ndoses)

power.contrast[replicate]=ifelse(pvalue<alpha,1,0)


fitemax = fitMod(dose, resp, data=dfData.combined, model="emax",  bnds = c(0.01, 25))

estimated.dose50[replicate]=doseforgivenmean(fitemax,-log(0.5))


estimated.ed50[replicate]=fitemax$coefs[3]


}

return(list(power.contrast=mean(power.contrast),conclude.doseeffect=power.contrast,estimated.dose50=estimated.dose50,estimated.ed50=es
}




#Function to find dose that gives target response assuming three-parameter emax
doseforgivenmean <- function(model, mu) {


  return((model$coefs[3]*(mu-model$coefs[1]))/(model$coefs[2]+model$coefs[1]-mu))
}


#Function for bootstrapping  #currently not modified to work with transformed outcome
dr50norm.boot <- function(data, indices) {
  y <- fit + e[indices]
  fitemaxboot = fitMod(data$dose, y, model="emax",  bnds = c(0.01, 50))
  est.parm = coef(fitemaxboot)
  ED50 = est.parm["ed50"] # extract the estimated ED50
  EMAX = est.parm["eMax"] # extract the estimated EMAX
  E0 = est.parm["e0"] # extract the estimated E0
  phi <- unname((50-E0)/EMAX)
  DoseR <- unname(phi*ED50/(1-phi))
  return(DoseR)
}

 #Scenario 1: large plateau effect, emax model
set.seed(1)
results.scenario1=simulation(nreplicates=10000,ed50 = 3)
print(c("Median ED50: ",median(results.scenario1$estimated.ed50,na.rm=T)))
print(c("Power: ",results.scenario1$power.contrast))

#Scenario 2: medium plateau effect, emax model
set.seed(1)
results.scenario2=simulation(maximumpercentagereduction = 60,ed50=1,nreplicates=10000 )
print(c("Median ED50: ",median(results.scenario2$estimated.ed50,na.rm=T)))
print(c("Power: ",results.scenario2$power.contrast))


#Scenario 3: medium plateau effect, emax model with ED50 equal to 3
set.seed(1)
results.scenario3=simulation(maximumpercentagereduction = 60,ed50=3,nreplicates=10000 )
print(c("Median ED50: ",median(results.scenario3$estimated.ed50,na.rm=T)))
print(c("Power: ",results.scenario3$power.contrast))


#Scenario 4: null:
set.seed(1)
results.scenario4=simulation(minimumpercentagereduction = 5,maximumpercentagereduction = 5,ed50=100,nreplicates=10000 )
print(c("Median ED50: ",median(results.scenario4$estimated.ed50,na.rm=T)))
print(c("Power: ",results.scenario4$power.contrast))
```
