# Supplementary material for: Graves-PCD: protocol for a randomised, dose-finding, adaptive trial of the plasma cell-depleting agent daratumumab in severe Graves’ disease
Source: BMJ Open. 2024 Jun 12;14(6):e079158. doi: 10.1136/bmjopen-2023-079158 (PMC11177693; doi:10.1136/bmjopen-2023-079158)
Supplement: Supplementary data [file bmjopen-2023-079158supp002.pdf]

Graves-PCD: Protocol for a randomised, dose-finding, adaptive trial of the plasma cell depleting agent daratumumab in severe Graves' disease

Supplementary Material 2

Table S2: Protocol Version History

Protocol version no.	Details of changes made
6.0	4. Trial Design- update to Figure 2<br>5. Trial Settings update<br>6.1. Update to inclusion criteria 2 and 4<br>7.2.1. Patient Identification- Addition of self-referral participant information<br>8.6. Dosage Schedule & Modifications- Clarification on IRRs<br>Appendix 3- Update to Infusion Rate Tables text and Infusion Related Reactions (IRR) concerning Grade 1-2 reactions management.
5.0	8.6 update to Dosage Schedule & Modifications<br>Appendix 3- 16.3.2 update to Infusion Rate Tables text
4.0	Update to Key Trial Contacts<br>Appendix 3- Update to Dose Banding Tables, Infusion Rate Table and Infusion Related Reactions (IRR)
3.0	Update to Key Trial Contacts<br>Typographical corrections<br>7.1.8 Storage of TRAb sample until data lock at visits 4, 7, 8 and 9<br>7.1.11 Addition of end of trial questions<br>7.7 Removal of reconfirmation of eligibility >6 weeks, addition of re-screening >3 months
2.0	2. Addition of rationale for choice of doses in stage 1<br>6.2 Update to exclusion criterion 6<br>7.1.5 Addition of photography for patients with thyroid eye disease<br>7.12 Removal of use of initials<br>8.3 and 9.3.1 Removal of reference to SmPC section 4.5<br>9.4 Addition of yellow card reporting for NIMPs<br>10.1.4 Clarification on criteria for safety assessment at interim analysis<br>16.3.3 Update to response to IRRs
1.0	Original version

Wolstenhulme F, *et al. BMJ Open* 2024; 14:e079158. doi: 10.1136/bmjopen-2023-079158
