# Supplementary material for: Graves-PCD: protocol for a randomised, dose-finding, adaptive trial of the plasma cell-depleting agent daratumumab in severe Graves’ disease
Source: BMJ Open. 2024 Jun 12;14(6):e079158. doi: 10.1136/bmjopen-2023-079158 (PMC11177693; doi:10.1136/bmjopen-2023-079158)
Supplement: Author's manuscript [file bmjopen-2023-079158.draft_revisions.pdf]

BMJ Open is committed to open peer review. As part of this commitment we make the peer review history of every article we publish publicly available.

When an article is published we post the peer reviewers' comments and the authors' responses online. We also post the versions of the paper that were used during peer review. These are the versions that the peer review comments apply to.

The versions of the paper that follow are the versions that were submitted during the peer review process. They are not the versions of record or the final published versions. They should not be cited or distributed as the published version of this manuscript.

BMJ Open is an open access journal and the full, final, typeset and author-corrected version of record of the manuscript is available on our site with no access controls, subscription charges or pay-per-view fees (http://bmjopen.bmj.com).

If you have any questions on BMJ Open's open peer review process please email info.bmjopen@bmj.com

BMJ Open

Graves-PCD: Protocol for a randomised, dose-finding, adaptive trial of the plasma cell depleting agent daratumumab in severe Graves' disease

Journal:	*BMJ Open*
Manuscript ID	bmjopen-2023-079158
Article Type:	Protocol
Date Submitted by the Author:	23-Aug-2023

Complete List of Authors:	Wolstenhulme, Faye; Newcastle University, Newcastle Clinical Trials Unit, 1-4 Claremont Terrace<br>Bibby, Irena; Newcastle University, Newcastle Clinical Trials Unit, 1-4 Claremont Terrace<br>Cole, Michael; Newcastle University, Population Health Sciences Institute, 4th Floor Ridley Building 1, Queen Victoria Road<br>Grixti, Lydia; Newcastle University, Translational and Clinical Research Institute, Faculty of Medical Sciences, Biomedicine West, International Centre for Life,<br>McGregor, Naomi; Newcastle University, Newcastle Clinical Trials Unit, 1-4 Claremont Terrace<br>Bradley, Penny; Newcastle Upon Tyne Hospitals NHS Foundation Trust, Pharmacy Clinical Trials<br>Maier, Rebecca; Newcastle University, Newcastle Clinical Trials Unit, 1-4 Claremont Terrace<br>Walker, Jenn; Newcastle University, Newcastle Clinical Trials Unit, 1-4 Claremont Terrace<br>Pearce, Simon H; Newcastle University, Translational and Clinical Research Institute, Faculty of Medical Sciences, Biomedicine West, International Centre for Life,<br>Wason, James; Newcastle University, Population Health Sciences Institute, Newcastle University, 4th Floor Ridley Building 1, Queen Victoria Road,

Keywords:	Patients, General endocrinology < DIABETES & ENDOCRINOLOGY, Thyroid disease < DIABETES & ENDOCRINOLOGY, Clinical Trial

SCHOLARONE™
Manuscripts

Graves-PCD: Protocol for a randomised, dose-finding, adaptive trial of the plasma cell depleting agent daratumumab in severe Graves' disease

Authors

Faye Wolstenhulme[1], Irena Bibby[1], Michael Cole[2], Lydia Grixti[3], Naomi McGregor[1], Penny Bradley[4], Rebecca Maier[1], Jenn Walker[1], Simon H Pearce[3], James Wason[2]

[1] Newcastle Clinical Trials Unit, Newcastle University, 1-4 Claremont Terrace, Newcastle upon Tyne, NE2 4AE

[2] Population Health Sciences Institute, Newcastle University, 4th Floor Ridley Building 1, Queen Victoria Road, Newcastle upon Tyne, NE1 7RU, UK

[3] Translational and Clinical Research Institute, Faculty of Medical Sciences, Biomedicine West, International Centre for Life, Newcastle University, Newcastle upon Tyne, NE1 3BZ

[4] Pharmacy Dept. Royal Victoria Infirmary, Queen Victoria Road, Newcastle upon Tyne, NE1 4LP

Correspondence to james.wason@newcastle.ac.uk

ORCiD IDs

FW: 0000-0002-4086-7931 IB: 0009-0007-3105-9864 MC: 0000-0003-3426-3717
NM: 0000-0002-5961-124X RM: 0000-0002-7350-3288 JWal: 0000-0002-7442-3060
SHP: 0000-0001-8384-8063, JWas: 0000-0002-4691-126X

Word count: Abstract 285, Text 3747, Tables 5.

For peer review only - http://bmjopen.bmj.com/site/about/guidelines.xhtml

ABSTRACT

Introduction: Severe Graves' disease is a life-changing condition with poor outcomes from currently available treatments. It is caused by directly pathogenic thyroid stimulating hormone (TSH) receptor stimulating antibodies (TRAb), which are secreted from plasma cells. The human anti-CD38 monoclonal antibody daratumumab was developed to target plasma cells which express high levels of CD38, and is currently licensed for treatment of the plasma cell malignancy, myeloma. However, it can also deplete benign plasma cells with the potential to reduce TRAb and alter the natural history of severe Graves' disease. This study aims to establish proof of concept that daratumumab has efficacy in severe Graves' disease patients and will provide important data to inform a choice of dosing regimen for subsequent trials.

Methods and Analysis: The Graves-PCD trial aims to determine if daratumumab modulates the humoral immune response in patients with severe Graves' disease, and if so, over what time period, and to find an optimal dose. It is a single blinded, randomised, dose-finding, adaptive trial using 4 different doses of daratumumab or placebo in 30 adult patients. Part 1 of the trial is dose-finding and, following an interim analysis; in part 2 the remaining patients will be randomised between the chosen dose(s) from the interim analysis or placebo. The primary outcome is the change in serum TRAb antibodies from baseline to 12 weeks.

Ethics and Dissemination

The trial received a favourable ethical opinion from London - Hampstead Research Ethics Committee (reference 21/LO/0449). The results of this trial will be disseminated at international meetings, in the peer-reviewed literature and through partner patient group newsletters and presentations at patient education events.

Registration

ISRCTN81162400

Funding information

This trial is funded by the UKRI Medical Research Council (MRC), ref MR/V005898/1

For peer review only

STRENGTHS AND LIMITATIONS OF THIS STUDY

- Current treatments for patients with severe Graves' disease are unsatisfactory and there is therefore significant unmet clinical need for new treatment
- Adaptive design allows efficient investigation of multiple doses
- The long-term remission of hyperthyroidism cannot be assessed in the 24 week follow-up period
- The duration of treatment or dosing intervals will not be tested in this study

For peer review only

INTRODUCTION

Graves' disease (autoimmune hyperthyroidism) affects around 3% of women and 0·5% of men, and most commonly presents in people in their 30s and 40s with a disproportionate burden of ill-health falling on working-age women.[1 2] Typical symptoms include weight loss, palpitations, heat intolerance, tremor, breathlessness, sweating, insomnia, loss of concentration and irritability. Around 40% of patients develop Thyroid Eye Disease (TED),[3] which can cause facial disfigurement as well as functional visual problems and even loss of sight. Quality of life in TED patients is poor, worse than for diabetes, and similar to patients with inflammatory bowel disease.[4] Rarely, Thyroid Dermopathy (Pretibial Myxoedema) may also occur leading to a brawny thickening of the skin on the lower legs and feet.[5] These latter 2 problems produce particularly distressing symptoms for a relatively young and active patient group. Around 10% of Graves' patients have a severe form of the disease, and experience severe thyrotoxicosis (serum FT4 ≥50pmol/l [upper limit of reference range 22pmol/l] or FT3≥15pmol [upper limit of reference range 6.8pmol/L]), failure of medical control of hyperthyroidism, large goitre, TED of sufficient severity to affect visual function or Thyroid Dermopathy. Current treatments for these patients with severe Graves' disease give unsatisfactory outcomes and are expensive, typically involving surgical thyroidectomy with or without several episodes of eye surgery.[6]

Conventional treatment of Graves' disease with antithyroid drugs (carbimazole, methimazole or propylthiouracil) leads to remission for only around 50% of patients, decreasing to less than 20% for patients with severe disease, and does not improve eye symptoms.[7 8] Other

For peer review only - http://bmjopen.bmj.com/site/about/guidelines.xhtml

treatment options of radio-active iodine or surgical thyroidectomy result in life-long hypothyroidism requiring levothyroxine therapy, which patients are often unwilling to accept.[9] Better treatment options are sorely needed for severe Graves' disease.

Severe Graves' disease is caused by high titres of directly pathogenic thyroid stimulating hormone receptor stimulating antibodies (TRAb), which are secreted from terminally differentiated B lymphocytes known as plasma cells.[10] Not only do TRAbs directly stimulate thyroid hormone over-production (leading to hyperthyroidism), they also cause thyrocyte hyperplasia leading to goitre, and bind to TSH receptors on the surface of orbital fibroblasts leading to mitogenic signalling that triggers and perpetuates eye disease. Failure of medical control and/or early relapse following conventional antithyroid drugs reflects the persistence of these long-lived, TRAb-secreting plasma cells in the secondary lymphoid tissues and bone marrow.

Both benign and malignant plasma cells express high levels of the cell-surface glycoprotein CD38. Daratumumab, a biological drug that targets CD38, was recently licenced for the plasma cell malignancy, myeloma.[11,12] This human anti-CD38 monoclonal antibody (mAb) has the potential to deplete plasma cells and therefore produce a rapid reduction in TRAb levels, which may alter the natural history of severe Graves' disease. This study aims to establish proof of concept that daratumumab has efficacy in severe Graves' disease patients and will provide important data to inform a choice of dosing regimen.
Tolbert et al. first reported the use of daratumumab in the treatment of benign, antibody-mediated haematological autoimmune disorders in 2016.[13] In this case a 19-year-old woman with autoimmune haemolytic anaemia (post stem cell transplant) was successfully treated with daratumumab and had an instant improvement in haemolysis. There are now numerous additional reports of daratumumab being repurposed to target benign disorders.[14]

Daratumumab is licenced for use in myeloma at a dose of 16mg/kg, however, patients with Graves' disease have several orders of magnitude fewer plasma cells and it is therefore expected that lower doses of drug will be active in this patient group with benign disease. To address this question the first stage of the study will be dose-finding. Notably, dose-finding trials in myeloma patients showed no difference in adverse event rates between doses of

For peer review only - http://bmjopen.bmj.com/site/about/guidelines.xhtml

8mg/kg and 16mg/kg, and no dose-limiting toxicity up to 24mg/kg.[11] Therefore, in order to define a signal for efficacy, this study will use 9mg/kg as the top dose in stage 1, with reducing concentrations (3mg/kg, 1mg/kg and 0.5mg/kg) along with placebo to determine the dose response in stage 1. As myeloma trials did not find increased adverse events or toxicities at higher doses, these four dose allocations will take place in parallel. Stage 2 of the study will randomise additional patients to one or more optimal doses of daratumumab, as selected from stage 1, or placebo to gain further information about real-life effect size, safety and tolerability.

METHODS AND ANALYSIS

This study protocol is based on the Standard Protocol Items: Recommendations for Interventional Trials (SPIRIT) 2013 statement.[15]

Sponsor and Trial Management

The trial Sponsor is the Newcastle upon Tyne Hospitals NHS Foundation Trust (Email: tnu-tr.sponsormanagement@nhs.net). Trial management is delegated to Newcastle Clinical Trials Unit, Newcastle University.

Objectives

The primary objective is to determine if daratumumab modulates the humoral immune response in patients with severe Graves' disease, with the primary outcome measure the change in serum TRAb antibodies from baseline to 12 weeks. Secondary objectives include determining the optimal dose of daratumumab, assessing safety, patient quality of life and impact on eye symptoms, thyroid function and goitre size (see Table 1).

Table 1: Objectives and Outcome Measures

	Objectives	Outcome Measures
**Primary**	To determine if daratumumab modulates the humoral immune response in Graves' disease patients	Change in serum TRAb antibodies from baseline to 12 weeks compared to change in placebo group
**Secondary**	To determine how fast daratumumab modulates the humoral immune response in Graves' disease patients	Change in serum TRAb antibodies from baseline to 2, 4, 6, 12 and 24 weeks

For peer review only - http://bmjopen.bmj.com/site/about/guidelines.xhtml

	To determine the optimal dose (or dose range) of daratumumab for Graves' disease patients	Dose-response curve for daratumumab against change in serum TRAb antibodies from baseline to 6 and 12 weeks
	To determine if daratumumab reduces thyroid hormone levels	Change in serum FT3 and FT4 from baseline to 2, 4, 6, 12 and 24 weeks
	To determine if daratumumab changes the time course of serum TSH	Change in serum TSH from baseline to 2, 4, 6, 12 and 24 weeks
	To determine if daratumumab changes thyroid size	Change in thyroid volume from baseline to 24 weeks measured by ultrasound
	To determine if daratumumab changes other thyroid autoantibodies	Change in serum ATPO and thyroglobulin antibodies from baseline to 6, 12 and 24 weeks
	To determine if daratumumab improves thyroid eye disease	Change in CAS, composite eye index and GOQoL score from baseline to 6, 12 and 24 weeks
	To determine if daratumumab improves thyroid symptom-related QoL	Change in ThyPRO39 score from baseline to 6, 12 and 24 weeks
	To determine if daratumumab is safe in this patient group	Change in serum immunoglobulins, specific antibodies including (SARS-CoV2) and blood count parameters from baseline to 6, 12 and 24 weeks.<br>Adverse Reactions to 24 weeks
**Experimental**	To determine if daratumumab changes lymphocyte/plasma cell transcriptomic markers	Analysis of blood plasma cell markers and mRNA signature
	To determine if daratumumab changes the lymphocyte subsets	Change in lymphocyte subsets (by FACS) from baseline to 6, 12 and 24 weeks

Trial Design

This is an adaptive, 2-stage randomised phase IIa, single centre clinical trial that will aim to recruit 30 patients with severe Graves' disease from secondary care at the Newcastle upon Tyne Hospitals NHS Foundation Trust. It is a single blinded trial in which participants will be blind to allocation.

Stage 1 is a dose-response study using 4 doses of daratumumab (9mg/kg, 3mg/kg, 1mg/kg, 0.5mg/kg) and a colourless, volume-matched placebo infusion in approximately 15 patients (i.e. five groups of n=3, randomised in a 1:1:1:1:1 ratio using permuted random blocks without stratification).

Following Stage 1, an interim analysis will be performed in order to select an optimal dose(s) of daratumumab for Stage 2. The dose selection will be based on an analysis of the reduction in TRAb antibody concentration and safety assessed at 12 weeks. In stage 2, the remaining

For peer review only - http://bmjopen.bmj.com/site/about/guidelines.xhtml

patients will be randomised between placebo and one or two chosen doses of daratumumab depending on results of the interim analysis. Patients in both stages of the study will have all the same assessments and will be followed up for 24 weeks, with the primary endpoint being measured at week 12.

Inclusion Criteria

1. Patients ≥18yrs old
2. Recent-onset Graves' disease (within 12 months) (defined as date of first thyroid function test showing hyperthyroidism (FT4 and TSH) in current episode).
3. TRAb antibody concentrations above 10U/L (on Roche or Brahms TRAb binding assays)
4. One or more of:
   - Pre-treatment severe hyperthyroidism (FT4 ≥50 pmol/L; or FT3 ≥15 pmol/l)
   - Persisting hyperthyroidism despite more than 12 weeks of antithyroid drug therapy (defined as FT3 above the upper limit of the reference range following 12 weeks of carbimazole treatment at a dose of 40mg or more daily (or equivalent dose of PTU))
   - Inflammatory thyroid eye disease (defined as clinical activity score, CAS≥3), or thyroid dermopathy
   - Large (visible) goitre (WHO grade III)
   - 2 or more relapses (3 episodes in total) despite completing 12 months or more of medical treatment on each occasion. Relapse is defined as FT3 above the upper limit of the local reference range.
5. For women of child-bearing potential, willing to use a highly effective contraceptive method during their participation in the trial.
6. Able to understand and speak sufficient English to complete trial procedures
7. Willing and able to provide informed consent prior to any trial procedures taking place

For peer review only

Exclusion Criteria

1. Previous thyroidectomy, or radioiodine treatment within 2 years
2. Pregnant or breastfeeding, or with a plan for pregnancy within 6 months
3. Previous shingles, known untreated cervical dysplasia, hepatitis B & C or HIV infection
4. Anaemia (Hb ≤ 100g/L), thrombocytopenia (≤75 x10^9/L) or neutropenia (≤1.0 x10^9/L)

For peer review only - http://bmjopen.bmj.com/site/about/guidelines.xhtml

5. Known chronic obstructive pulmonary disease (COPD) (defined as a forced expiratory volume [FEV] in 1 second <60% of predicted normal), persistent asthma, or a history of asthma within the last 2 years (intermittent asthma without hospitalisation is allowed)
6. Any significant physical or mental health condition that impacts the safety of the intervention, the interpretation of thyroid function or the ability of a participant to attend for intervention and safety monitoring, eg. major cardiorespiratory disease, renal or hepatic failure, pancreatitis, cancer undergoing active treatment (excluding non-melanoma skin cancer), untreated chronic infection including TB, psychosis, depression impairing Activities of Daily Living
7. Current use of immunosuppressive therapy for thyroid eye disease or other conditions (within 3 months)
8. Current or previous participation in a CTIMP research study within 4 months
9. Hypersensitivity or anaphylactic reaction to previous monoclonal antibody treatments or methylprednisolone
10. Inability, in the opinion of the investigator, to be able to complete the clinical trial visits or procedures.

Risk Assessment

Daratumumab is a licenced product for myeloma patients and the main risk is that it will have a different side effect profile in patients with benign disease. The typical risks of daratumumab when used for myeloma include transient infusion reactions and haematological dyscrasias, the latter being accounted for by the bone marrow suppression in myeloma patients and leading to a small risk of infection (≤5%).

Daratumumab interferes with urgent blood cross matching and all patients will have a Coombs test and blood group and transfusion antibody screen prior to administration of daratumumab, as well as receiving an information card in case blood transfusion is needed. There is a potential for reactivation of latent viral illness, and so all participants will be screened for hepatitis B, C and HIV infection, and participants with previous shingles or current untreated cervical dysplasia will be excluded. There are no specific risks demonstrated in the Graves' disease population who will be younger and in better overall health than the patient group who currently receive daratumumab for myeloma.

For peer review only - http://bmjopen.bmj.com/site/about/guidelines.xhtml

Participants will be offered a course of SARS-CoV2 vaccination prior to treatment with IMP. If loss of immunity to specific pathogens is detected at the end of the study, the participants may be offered repeat vaccination for tetanus, pneumococcus and SARS-CoV2, where clinically appropriate. Throughout their participation in the study, patients will continue their regular "background" medication for treatment of thyrotoxicosis (i.e. antithyroid drugs, with or without levothyroxine) as prescribed by their clinician as per usual clinical care and will be carefully monitored with regular blood tests throughout the study.

Patient Identification, Consent and Screening

Potential participants with severe Graves' disease within secondary care will be identified and approached in person, by post or email with a trial participant information sheet (PIS) by one of the clinical team responsible for their usual management. Potential participants will also be identified and referred by local secondary care Patient Identification Centre sites, or can self-refer to the trial via adverts through organisations such as the British Thyroid Foundation (BTF). Consent discussions and written informed consent will take place in person at a research specific clinic appointment, with the principal investigator (PI) or alternative member of the research team who is medically qualified and appropriately trained. For women of child-bearing potential, consent discussions will include the need to use a highly effective form of contraception (as listed on the PIS) for the duration of their participation in the trial. Consent discussions will be documented in medical notes.

Following written informed consent, eligibility for the trial will be assessed by:

- Demographics and medical history including medication history
- Full external medical examination, including weight, heart (including resting pulse rate and 12 lead ECG), chest, abdomen, goitre size assessment, skin, eyes and neurological examination
- Blood tests (FBC, U&E, LFT, HBsAg, HCV antibodies, HIV, Bone chemistry, TRAb antibodies, serum FT4, FT3, TSH, serum IgGAM, Coombs, blood group and red cell phenotyping)
- A urine sample pregnancy test for all female participants of child-bearing potential (to be repeated where there is >14 days between screening and baseline visits)

The trial screening log will include reasons for screen fails and any reasons given for declining the trial.

For peer review only - http://bmjopen.bmj.com/site/about/guidelines.xhtml

Randomisation

Randomisation will be performed by delegated and trained members of the site research team using the online system “Sealed Envelope”; a central, secure, 24-hour web-based randomisation system. In both stages, the allocation sequence will be computer-generated using a random permuted block design. Patients will be randomly allocated equally to the available daratumumab doses or to a matched placebo; for example, if 2 doses of daratumumab plus placebo are to be used, the allocation would be 1:1:1. There are no stratification factors.

In Stage 1, patients will be allocated to receive a dose of daratumumab (9, 3, 1, 0.5mg/Kg) or matched placebo. In Stage 2, patients will be allocated to receive daratumumab (one or two dose levels as determined by the interim analysis), or matched placebo.

Intervention

Each participant will receive 2 infusions, on day 0 and day 14 (+/-3 days). The prescribed daratumumab dose will be prepared in a 500 or 1000ml bag of sodium chloride 0.9%, using the most recent available participant weight to calculate banded dose (see Table 2 for volume and infusion rate). Each participant will be prescribed the same banded dose for both infusions, using the same weight for both. Where placebo is allocated, a volume-matched bag of sodium chloride 0.9% will be used. All participants will be pre-medicated with three non-investigational medicinal products (NIMPs), paracetamol (1g po), methylprednisolone (100mg IV) and chlorphenamine (10mg IV) at least 30 minutes prior to their allocated infusion.

Table 2: Infusion Rate Table

Time (Hr:Min)	Rate (mls /Hr)	Total volume at end of time (ml)
**Stage 1 (1000mls)**		
1	80	80
2	160	240
3	240	480
4	320	800
4:40	320	1000
Stage 2 rate A (1000mls)		
1	50	50
2	100	150
3	150	300

For peer review only

4	200	500
5	200	700
6	200	900
6:30	200	1000
Stage 2 rate B (500mls)		
1	50	50
2	100	150
3	150	300
4	200	500

Table 2: The maximum infusion rate of daratumumab was determined by the recommendation of the SmPC for daratumamab (DARZALEX 20 mg/mL concentrate for solution for infusion). For Stage 1, all participants will receive the IMP/placebo diluted in 1000mls sodium chloride 0.9% for both infusions. For stage 2, some patients may receive higher doses of daratumumab, so a more prolonged first infusion will be given to all participants. If participants have no IRRs during their first dose in stage 2, then the second dose may be given in 500mls sodium chloride 0.9% with a faster infusion rate, in line with SmPC recommendations.

For peer review only

Vital signs (pulse rate, blood pressure and temperature) will be recorded prior to the start of each infusion and every 15 minutes during the course of the infusion, and for 1 hour afterwards. Infusion related reactions will be addressed as shown in Table 3, and documented as an adverse event. Infusion charts will be completed to document compliance with prescribed dose. Participants will be followed up by phone approximately 2 days after each infusion visit to check for any adverse events. After the day 14 visit, no further IMP will be administered. Patients will continue their regular medications, including antithyroid drugs and betablockers throughout the study, as deemed appropriate by the clinical team.

Table 3: Infusion Related Reactions

**Grade 4 reactions**	Life-threatening	Stop and permanently discontinue infusion.
**Grade 3 reactions**	Severe, including:<br>• Tachycardia ≥120BPM or drop in SBP ≥30mmHg over pre-infusion with symptoms<br>• Rise in SBP ≥30mmHg over pre-infusion or tachypnoea ≥24/min<br>• Hypoxia SaO2 <92%	Stop infusion until observations improve and restart at 50% of the infusion rate at which IRR occurred. If the patient does not experience additional symptoms, infusion rate escalation may be resumed at increments and intervals as appropriate. The procedure above should be repeated in the event of recurrence of Grade 3 symptoms. After the infusion re-start attempts, the IMP treatment will be

		permanently discontinued upon the third occurrence of a Grade 3 or greater IRR.
**Grade 1-2 reactions**	Mild to moderate, including:<br>• nasal congestion<br>• cough<br>• chills<br>• throat irritation<br>• vomiting<br>• nausea	Stop infusion in case of clinically significant reaction only until observations improve and restart at 50% of the infusion rate at which IRR occurred. If the patient does not experience any further IRR symptoms, infusion rate escalation may be resumed at increments and intervals as clinically appropriate up to the maximum rate. Infusion does not need to be stopped if IRR is not clinically significant.

Assessments

Follow up visits will take place at weeks 4, 6, 12 and 24 (+/- 1 week); the details of assessments are provided in Table 4. At each visit, concomitant medications will be recorded, an adverse event check will be performed, compliance with anti-thyroid medication checked and full blood count and thyroid function (TSH, FT4, FT3 and TRAb) will be assessed. Other assessments include goitre size assessment, eye exam, patient reported quality of life measures (GOQoL[4] and ThyPRO[16]), specific antibody tests and other safety bloods (FBC, LFT). At the end of their participation in the trial, participants will be asked their opinion on which treatment allocation they received, and if they would recommend their treatment to a friend with the same condition. In addition, we plan to undertake exploratory analyses of lymphocyte subsets determined by flow cytometry and transcriptomic markers of plasma cells using qRT-PCR.[17]

For peer review only

Table 4: Schedule of Assessments

**Assessment/Activity**	**Visit 1**	**Visits 2 & 3**	**Visit 4**	**Visit 5**	**Visit 6**	**Visit 7**	**Visit 8**	**Visit 9**
			**Day 0**	**Day 14 (+/-3 days)**	**4 weeks (+/- 1 week)**	**6 weeks (+/- 1 week)**	**12 weeks (+/- 1 week)**	**24 weeks (+/- 1 week)**
Written Informed Consent	X							
Demographics & medical history	X							
Physical exam	X							
12-lead ECG	X							
Eye exam (CAS)	X							
HBsAG, HCV antibodies, HIV	X							
Bone chem	X							

Assessment/Activity	Visit 1	Visits 2 & 3	Visit 4	Visit 5	Visit 6	Visit 7	Visit 8	Visit 9
Coombs, blood group, RBC phenotyping	X							
Pregnancy testing (females)	X						X	
Weight	X	X	X	X	X	X	X	X
Resting pulse rate	X		X	X	X	X	X	X
Goitre size assessment	X		X			X	X	X
FBC	X		X	X	X	X	X	X
TSH, FT4, FT3, TRAb	X		X	X	X	X	X	X
U&E, LFT	X		X	X	X	X		
IgGAM	X		X			X	X	X
Height			X					
Eye exam (composite eye index)			X			X	X	X
Thyroid volume USS			X					X
QoL questionnaires (GOQoL, ThyPRO)			X			X	X	X
TgAb, ATPO			X			X	X	X
Specific antibodies			X				X	X
Exploratory analysis sample			X			X	X	X
Vital signs			X	X				
Compliance with ATD			X	X	X	X	X	X
Concomitant medication		X	X	X	X	X	X	X
Adverse event reporting		X	X	X	X	X	X	X
Follow-up phone call			X	X				
End of trial questions								X

For peer review only

Discontinuation of Treatment and Withdrawal

Participants who have completed one or more daratumumab/placebo infusions may remain on the trial but choose to discontinue treatment. These participants will continue to take part in the trial assessments as per the trial schedule of events. Alternatively, an investigator may discontinue a participant from receiving trial treatment at any time (until administration of second infusion is completed), if the investigator considers it necessary for any reason. Participants who withdraw from the trial after receiving IMP (full or part dose) will not be replaced. Participants who withdraw before IMP is given will be replaced.

Safety Reporting

Adverse events (AEs) will be documented in patient medical notes -before entry into the trial database. AEs judged by the investigator responsible for patient care as consistent with the usual clinical pattern for patients with severe Graves' disease (e.g. Hyperthyroidism, hypothyroidism, tachycardia, bradycardia) will not be reported. Disease progression will be recorded as an AE only if it is more severe than expected in the trial population. AEs will be

coded according to MedDRA classifications. All AEs meeting the criteria for seriousness (SAEs) will be reported by the investigator to the trial Sponsor within 24 hours of awareness, and suspected unexpected serious adverse reactions will be reported to the MHRA and research ethics committee (REC) by the trial Sponsor in line with regulatory reporting timelines.

End of Trial

The end of the trial is defined as last patient last visit (LPLV). Treatment allocation will be revealed to all participants (and their GP/referring clinician where applicable) by letter at the end of the trial, with contact details to discuss if required. At the end of their participation in the study participants will return to standard NHS care.

Payment

No payment will be made to participants for taking part in this trial, although reasonable travel expense reimbursement including taxis will be made for trial visits.

Data Management

The Clinical Data Management System (CDMS) used to capture trial data is Red Pill, supplied by Sealed Envelope. Users have individual password protected user accounts appropriate to their trial duties (e.g. randomisation, data entry, monitor). Patients are identified on the CDMS using a unique participant ID. Data monitoring will include a mixture of central, off-site and on-site approaches and will be outlined in a risk-based monitoring plan prior to the start of the trial. The trial specific Data Management Plan will include details of how the validity and quality of data will be monitored and managed. As far as possible, validations will be built into the CDMS and additional manual validations will be detailed in the Data Validation Plan. Trial monitoring will be performed by NCTU as per the monitoring plan and the trial is also liable to audit by the trial Sponsor and regulator.

Until publication of the trial results, access to the full dataset will be restricted to the Trial Management Group (TMG) and to authors of the publication. Requests for anonymised data should be directed to the lead author.

Analysis

For peer review only

For peer review only - http://bmjopen.bmj.com/site/about/guidelines.xhtml

The primary analysis will assess whether there is a significant dose-response using percentage reduction in serum TRAb concentration from baseline to 12 weeks using the intention-to-treat (ITT) population (participants analysed according to randomisation allocation). To do this we will calculate a contrast-based test statistic at a one-sided 5% type I error rate.[18] If this test statistic is significant, we will test whether there is a mean difference in the outcome between individual doses and placebo using a suitable linear model. We will also perform a simple test of all placebo participants vs all daratumumab patients using a linear model that adjusts for presence of eye disease. We will also fit quadratic and three-parameter Emax models to assess dose-response.[19] This analysis will inform selection of the most suitable dose for subsequent trials. Exploratory analyses using subgroups of patients including by gender, smoking status, presence of TED and change in serum TRAb will be undertaken.

Interim Analyses and Criteria for the Premature Termination of the Trial

An interim analysis of dose-response will be conducted after 15 patients have provided 12-week follow-up data. To assess efficacy, a three-parameter Emax model will be fitted to the 12-week TRAb log-percentage change using the DoseFinding package in R[20] in the dose-response population (participants analysed according to the amount of daratumumab received). This model is used to estimate the plateau effect and i) the dose that gives a mean reduction of 50% and ii) the dose that gives 90% of the plateau effect. If the estimated plateau effect corresponds to >50% reduction in TRAb, the trial will continue with placebo and doses (i) and (ii) defined above. If the estimated plateau corresponds to less than 25% reduction in TRAb, the trial will terminate early for lack of promising dose-response unless the DMC agree that there has been an unequivocal improvement in serum thyroid hormone measurements in the active IMP vs placebo groups; if it is between 25%-50% reduction, the trial will continue with doses of 9mg/kg and a higher daratumumab dose (e.g. 12 or 16mg/Kg as determined by safety data), plus placebo. Safety information will be taken into consideration in deciding stage 2 dose(s) and could over-ride the model-based dose recommendations.

Statistical Size Calculations

A 50% or more reduction in TRAb antibody concentration from baseline within 12 weeks of intervention was chosen as biologically plausible and clinically important. An observational study of milder Graves' disease patients showed a mean change in TRAb concentration of 2%

For peer review only - http://bmjopen.bmj.com/site/about/guidelines.xhtml

over 6 weeks, with an SD of 15%. Assuming: a standard deviation of 25%, to account for the outcome being measured at 12 weeks; a 5% reduction in placebo treated subjects; an Emax dose-response; a plateau 60% reduction in TRAb concentration over 12 weeks with active IMP; and 1mg/kg dose giving half of the total, 27 participants will give over 90% power (5% one-sided type I error) to conclude there is a dose response relationship. In order to allow for 10% drop out, we will plan to recruit 30 participants.

Power was verified through simulations assuming the logarithm of the percentage reduction to be normally distributed with SD of 0.5 (equivalent to 25% SD when the mean is 50% reduction). The power/type I error rate for a range of scenarios is given in Table 5.

Table 5: Power/type 1 error rate calculations

Scenario	Probability to conclude dose-response (10000 replicates)
1 – plateau of 80% reduction ED50 =3mg/kg	>99%
2 – plateau of 60% reduction, ED50=1mg/kg	92%
3 – plateau of 60% reduction, ED50=3mg/kg	83%
4 – null scenario	3.2%

Trial Oversight

The TMG will meet approximately monthly throughout the trial, and includes the CI, statisticians, Newcastle Clinical Trials Unit staff, pharmacy and Sponsor representatives. Oversight is provided by an independent Data Monitoring Committee who will meet at the start of the trial, at the interim analysis point and on an ad hoc basis throughout, as well as a Trial Steering Committee (TSC) who will generally meet following a DMC meeting. The terms of reference and composition of these committees are outlined in their respective charters.

Protocol Amendments

NCTU will submit amendments to the HRA and MHRA/REC (as appropriate) as well as informing ISRCTN, trial recruiters, investigators and the relevant R&D department of changes. A list of version changes will be kept in the trial protocol appendix.

Trial Status

For peer review only

For peer review only - http://bmjopen.bmj.com/site/about/guidelines.xhtml

This manuscript is based on protocol version 6.0 dated 18 May 2023. The Graves-PCD trial opened to recruitment on 29 September 2021. At the time of writing, stage 2 of the trial is open to recruitment.

Patient and Public Involvement

Patients were involved in the design of the trial via the British Thyroid Foundation (www.btf-thyroid.org), and lay members will have oversight and input during the trial and dissemination as members of the TSC.

ETHICS AND DISSEMINATION

The trial received a favourable ethical opinion from London - Hampstead Research Ethics Committee (reference 21/LO/0449). The results of this trial will be disseminated at international meetings, in the peer-reviewed literature and through partner patient group newsletters and presentations at patient education events.

For peer review only

For peer review only - http://bmjopen.bmj.com/site/about/guidelines.xhtml

REFERENCES

1. Nyström HF, Jansson S, Berg G. Incidence rate and clinical features of hyperthyroidism in a long-term iodine sufficient area of Sweden (Gothenburg) 2003-2005. *Clin Endocrinol (Oxf)* 2013;**78**(5):768-76.
2. Vanderpump MP. The epidemiology of thyroid disease. *Br Med Bull* 2011;**99**:39-51.
3. Chin YH, Ng CH, Lee MH, et al. Prevalence of thyroid eye disease in Graves' disease: A meta-analysis and systematic review. *Clinical Endocrinology* 2020;**93**(4):363-74.
4. Gerding MN, Terwee CB, Dekker FW, et al. Quality of life in patients with Graves' ophthalmopathy is markedly decreased: measurement by the medical outcomes study instrument. *Thyroid* 1997;**7**(6):885-9.
5. Fatourechi V, Pajouhi M, Fransway AF. Dermopathy of Graves disease (pretibial myxedema). Review of 150 cases. *Medicine (Baltimore)* 1994;**73**(1):1-7.
6. Estcourt S, Hickey J, Perros P, et al. The patient experience of services for thyroid eye disease in the United Kingdom: results of a nationwide survey. *Eur J Endocrinol* 2009;**161**(3):483-7.
7. Struja T, Fehlberg H, Kutz A, et al. Can we predict relapse in Graves' disease? Results from a systematic review and meta-analysis. *Eur J Endocrinol* 2017;**176**(1):87-97.
8. Eckstein AK, Plicht M, Lax H, et al. Thyrotropin receptor autoantibodies are independent risk factors for Graves' ophthalmopathy and help to predict severity and outcome of the disease. *J Clin Endocrinol Metab* 2006;**91**(9):3464-70.
9. van Kinschot CMJ, Soekhai VR, de Bekker-Grob EW, et al. Preferences of patients and clinicians for treatment of Graves' disease: a discrete choice experiment. *Eur J Endocrinol* 2021;**184**(6):803-812.
10. Smith TJ, Hegedüs L. Graves' Disease. *N Engl J Med* 2016;**375**(16):1552-65.
11. Lokhorst HM, Plesner T, Laubach JP, et al. Targeting CD38 with Daratumumab Monotherapy in Multiple Myeloma. *New England Journal of Medicine* 2015;**373**(13):1207-19.
12. Lonial S, Weiss BM, Usmani SZ, et al. Daratumumab monotherapy in patients with treatment-refractory multiple myeloma (SIRIUS): an open-label, randomised, phase 2 trial. *Lancet* 2016;**387**(10027):1551-60.
13. Tolbert VP, Goldsby R, Huang J, et al. Daratumumab Is Effective in the Treatment of Refractory Post-Transplant Autoimmune Hemolytic Anemia: A Pediatric Case Report. *Blood* 2016;**128**(22):4819-19.
14. Ostendorf L, Burns M, Durek P, et al. Targeting CD38 with Daratumumab in Refractory Systemic Lupus Erythematosus. *New England Journal of Medicine* 2020;**383**(12):1149-55.
15. Chan AW, Tetzlaff JM, Gøtzsche PC, et al. SPIRIT 2013 explanation and elaboration: guidance for protocols of clinical trials. BMJ. 2013 Jan 8;346:e7586. doi: 10.1136/bmj.e7586.
16. Watt T, Bjorner JB, Groenvold M, et al. Development of a Short Version of the Thyroid-Related Patient-Reported Outcome ThyPRO. *Thyroid* 2015;**25**(10):1069-79.
17. Streicher K, Morehouse CA, Groves CJ, et al. The plasma cell signature in autoimmune disease. *Arthritis Rheumatol* 2014; **66**(1):173-84.
18. Thomas N. Understanding MCP-MOD dose finding as a method based on linear regression. *Stat Med* 2017;**36**(27):4401-13.
19. Macdougall J. Analysis of Dose–Response Studies—Emax Model. In: Ting N, ed. Dose Finding in Drug Development. New York, NY: Springer New York, 2006:127-45.

For peer review only

20. Pinheiro J, Bornkamp B, Glimm E, et al. Model-based dose finding under model uncertainty using general parametric models. *Statistics in Medicine* 2014;**33**(10):1646-61.

ACKNOWLEDGEMENTS

We would like to acknowledge the ongoing support provided by the British Thyroid Foundation. We are grateful to Prof Ruth Andrew of Edinburgh University for advice concerning dose choices and to Dr. Catherine Stroud of Newcastle Hospitals NHS Trust for immunological safety advice.

AUTHORS' CONTRIBUTIONS

SHP had the original idea for the trial. The trial protocol was developed by SP, with substantial input and revision by FW, JWas, MC, RM, JWal and PB. IB and NM also made changes to the protocol at various stages of development. The trial protocol manuscript was primarily drafted by FW, with substantial input from SHP and JWas. All other coauthors approved the final version of the manuscript.

COMPETING INTERESTS STATEMENT.

SHP has received speaker fees from Merck, IBSA, and consulted for Apitope/Worg and Immunovant/Roivant. JWas has consulted for Worg. No other author has conflicts of interest to declare.

For peer review only

For peer review only - http://bmjopen.bmj.com/site/about/guidelines.xhtml

SPIRIT 2013 Checklist: Recommended items to address in a clinical trial protocol and related documents*

Section/item	Item No	Description
**Administrative information**		
Title	1	Descriptive title identifying the study design, population, interventions, and, if applicable, trial acronym
Trial registration	2a	Trial identifier and registry name. If not yet registered, name of intended registry
	2b	All items from the World Health Organization Trial Registration Data Set
Protocol version	3	Date and version identifier
Funding	4	Sources and types of financial, material, and other support
Roles and responsibilities	5a	Names, affiliations, and roles of protocol contributors
	5b	Name and contact information for the trial sponsor
	5c	Role of study sponsor and funders, if any, in study design; collection, management, analysis, and interpretation of data; writing of the report; and the decision to submit the report for publication, including whether they will have ultimate authority over any of these activities
	5d	Composition, roles, and responsibilities of the coordinating centre, steering committee, endpoint adjudication committee, data management team, and other individuals or groups overseeing the trial, if applicable (see Item 21a for data monitoring committee)
**Introduction**		
Background and rationale	6a	Description of research question and justification for undertaking the trial, including summary of relevant studies (published and unpublished) examining benefits and harms for each intervention
	6b	Explanation for choice of comparators
Objectives	7	Specific objectives or hypotheses
Trial design	8	Description of trial design including type of trial (eg, parallel group, crossover, factorial, single group), allocation ratio, and framework (eg, superiority, equivalence, noninferiority, exploratory)

For peer review only

Methods: Participants, interventions, and outcomes

Study setting	9	Description of study settings (eg, community clinic, academic hospital) and list of countries where data will be collected. Reference to where list of study sites can be obtained
Eligibility criteria	10	Inclusion and exclusion criteria for participants. If applicable, eligibility criteria for study centres and individuals who will perform the interventions (eg, surgeons, psychotherapists)
Interventions	11a	Interventions for each group with sufficient detail to allow replication, including how and when they will be administered
	11b	Criteria for discontinuing or modifying allocated interventions for a given trial participant (eg, drug dose change in response to harms, participant request, or improving/worsening disease)
	11c	Strategies to improve adherence to intervention protocols, and any procedures for monitoring adherence (eg, drug tablet return, laboratory tests)
	11d	Relevant concomitant care and interventions that are permitted or prohibited during the trial
Outcomes	12	Primary, secondary, and other outcomes, including the specific measurement variable (eg, systolic blood pressure), analysis metric (eg, change from baseline, final value, time to event), method of aggregation (eg, median, proportion), and time point for each outcome. Explanation of the clinical relevance of chosen efficacy and harm outcomes is strongly recommended
Participant timeline	13	Time schedule of enrolment, interventions (including any run-ins and washouts), assessments, and visits for participants. A schematic diagram is highly recommended (see Figure)
Sample size	14	Estimated number of participants needed to achieve study objectives and how it was determined, including clinical and statistical assumptions supporting any sample size calculations
Recruitment	15	Strategies for achieving adequate participant enrolment to reach target sample size

Methods: Assignment of interventions (for controlled trials)

Allocation:

Sequence generation	16a	Method of generating the allocation sequence (eg, computer-generated random numbers), and list of any factors for stratification. To reduce predictability of a random sequence, details of any planned restriction (eg, blocking) should be provided in a separate document that is unavailable to those who enrol participants or assign interventions

For peer review only - http://bmjopen.bmj.com/site/about/guidelines.xhtml

Allocation concealment mechanism	16b	Mechanism of implementing the allocation sequence (eg, central telephone; sequentially numbered, opaque, sealed envelopes), describing any steps to conceal the sequence until interventions are assigned
Implementation	16c	Who will generate the allocation sequence, who will enrol participants, and who will assign participants to interventions
Blinding (masking)	17a	Who will be blinded after assignment to interventions (eg, trial participants, care providers, outcome assessors, data analysts), and how
	17b	If blinded, circumstances under which unblinding is permissible, and procedure for revealing a participant's allocated intervention during the trial
**Methods: Data collection, management, and analysis**		
Data collection methods	18a	Plans for assessment and collection of outcome, baseline, and other trial data, including any related processes to promote data quality (eg, duplicate measurements, training of assessors) and a description of study instruments (eg, questionnaires, laboratory tests) along with their reliability and validity, if known. Reference to where data collection forms can be found, if not in the protocol
	18b	Plans to promote participant retention and complete follow-up, including list of any outcome data to be collected for participants who discontinue or deviate from intervention protocols
Data management	19	Plans for data entry, coding, security, and storage, including any related processes to promote data quality (eg, double data entry; range checks for data values). Reference to where details of data management procedures can be found, if not in the protocol
Statistical methods	20a	Statistical methods for analysing primary and secondary outcomes. Reference to where other details of the statistical analysis plan can be found, if not in the protocol
	20b	Methods for any additional analyses (eg, subgroup and adjusted analyses)
	20c	Definition of analysis population relating to protocol non-adherence (eg, as randomised analysis), and any statistical methods to handle missing data (eg, multiple imputation)
**Methods: Monitoring**		
Data monitoring	21a	Composition of data monitoring committee (DMC); summary of its role and reporting structure; statement of whether it is independent from the sponsor and competing interests; and reference to where further details about its charter can be found, if not in the protocol. Alternatively, an explanation of why a DMC is not needed

For peer review only - http://bmjopen.bmj.com/site/about/guidelines.xhtml

	21b	Description of any interim analyses and stopping guidelines, including who will have access to these interim results and make the final decision to terminate the trial
Harms	22	Plans for collecting, assessing, reporting, and managing solicited and spontaneously reported adverse events and other unintended effects of trial interventions or trial conduct
Auditing	23	Frequency and procedures for auditing trial conduct, if any, and whether the process will be independent from investigators and the sponsor
**Ethics and dissemination**		
Research ethics approval	24	Plans for seeking research ethics committee/institutional review board (REC/IRB) approval
Protocol amendments	25	Plans for communicating important protocol modifications (eg, changes to eligibility criteria, outcomes, analyses) to relevant parties (eg, investigators, REC/IRBs, trial participants, trial registries, journals, regulators)
Consent or assent	26a	Who will obtain informed consent or assent from potential trial participants or authorised surrogates, and how (see Item 32)
	26b	Additional consent provisions for collection and use of participant data and biological specimens in ancillary studies, if applicable
Confidentiality	27	How personal information about potential and enrolled participants will be collected, shared, and maintained in order to protect confidentiality before, during, and after the trial
Declaration of interests	28	Financial and other competing interests for principal investigators for the overall trial and each study site
Access to data	29	Statement of who will have access to the final trial dataset, and disclosure of contractual agreements that limit such access for investigators
Ancillary and post-trial care	30	Provisions, if any, for ancillary and post-trial care, and for compensation to those who suffer harm from trial participation
Dissemination policy	31a	Plans for investigators and sponsor to communicate trial results to participants, healthcare professionals, the public, and other relevant groups (eg, via publication, reporting in results databases, or other data sharing arrangements), including any publication restrictions
	31b	Authorship eligibility guidelines and any intended use of professional writers
	31c	Plans, if any, for granting public access to the full protocol, participant-level dataset, and statistical code

For peer review only

Appendices

Informed consent materials	32	Model consent form and other related documentation given to participants and authorised surrogates
Biological specimens	33	Plans for collection, laboratory evaluation, and storage of biological specimens for genetic or molecular analysis in the current trial and for future use in ancillary studies, if applicable

*It is strongly recommended that this checklist be read in conjunction with the SPIRIT 2013 Explanation & Elaboration for important clarification on the items. Amendments to the protocol should be tracked and dated. The SPIRIT checklist is copyrighted by the SPIRIT Group under the Creative Commons "Attribution-NonCommercial-NoDerivs 3.0 Unported" license.

For peer review only

BMJ Open

Graves-PCD: Protocol for a randomised, dose-finding, adaptive trial of the plasma cell depleting agent daratumumab in severe Graves' disease

Journal:	*BMJ Open*
Manuscript ID	bmjopen-2023-079158.R1
Article Type:	Protocol
Date Submitted by the Author:	01-Mar-2024
Complete List of Authors:	Wolstenhulme, Faye; Newcastle University, Newcastle Clinical Trials Unit, 1-4 Claremont Terrace<br>Bibby, Irena; Newcastle University, Newcastle Clinical Trials Unit, 1-4 Claremont Terrace<br>Cole, Michael; Newcastle University, Population Health Sciences Institute, 4th Floor Ridley Building 1, Queen Victoria Road<br>Grixti, Lydia; Newcastle University, Translational and Clinical Research Institute, Faculty of Medical Sciences, Biomedicine West, International Centre for Life,<br>McGregor, Naomi; Newcastle University, Newcastle Clinical Trials Unit, 1-4 Claremont Terrace<br>Bradley, Penny; Newcastle Upon Tyne Hospitals NHS Foundation Trust, Pharmacy Clinical Trials<br>Maier, Rebecca; Newcastle University, Newcastle Clinical Trials Unit, 1-4 Claremont Terrace<br>Walker, Jenn; Newcastle University, Newcastle Clinical Trials Unit, 1-4 Claremont Terrace<br>Pearce, Simon H; Newcastle University, Translational and Clinical Research Institute, Faculty of Medical Sciences, Biomedicine West, International Centre for Life,<br>Wason, James; Newcastle University, Population Health Sciences Institute, Newcastle University, 4th Floor Ridley Building 1, Queen Victoria Road,
**Primary Subject Heading**:	Diabetes and endocrinology
Secondary Subject Heading:	Research methods
Keywords:	Patients, General endocrinology < DIABETES & ENDOCRINOLOGY, Thyroid disease < DIABETES & ENDOCRINOLOGY, Clinical Trial

SCHOLARONE™
Manuscripts

Graves-PCD: Protocol for a randomised, dose-finding, adaptive trial of the plasma cell depleting agent daratumumab in severe Graves' disease

Authors

Faye Wolstenhulme[1], Irena Bibby[1], Michael Cole[2], Lydia Grixti[3], Naomi McGregor[1], Penny Bradley[4], Rebecca Maier[1], Jenn Walker[1], Simon H Pearce[3], James Wason[2]

[1] Newcastle Clinical Trials Unit, Newcastle University, 1-4 Claremont Terrace, Newcastle upon Tyne, NE2 4AE

[2] Population Health Sciences Institute, Newcastle University, 4th Floor Ridley Building 1, Queen Victoria Road, Newcastle upon Tyne, NE1 7RU, UK

[3] Translational and Clinical Research Institute, Faculty of Medical Sciences, Biomedicine West, International Centre for Life, Newcastle University, Newcastle upon Tyne, NE1 3BZ

[4] Pharmacy Dept. Royal Victoria Infirmary, Queen Victoria Road, Newcastle upon Tyne, NE1 4LP

Correspondence to james.wason@newcastle.ac.uk

ORCiD IDs

FW: 0000-0002-4086-7931 IB: 0009-0007-3105-9864 MC: 0000-0003-3426-3717
NM: 0000-0002-5961-124X RM: 0000-0002-7350-3288 JWal: 0000-0002-7442-3060
SHP: 0000-0001-8384-8063, JWas: 0000-0002-4691-126X

Word count: Abstract 285, Text 3779, Tables 5, Figures 1.

For peer review only

ABSTRACT

Introduction: Severe Graves' disease is a life-changing condition with poor outcomes from currently available treatments. It is caused by directly pathogenic thyroid stimulating hormone (TSH) receptor stimulating antibodies (TRAb), which are secreted from plasma cells. The human anti-CD38 monoclonal antibody daratumumab was developed to target plasma cells which express high levels of CD38, and is currently licensed for treatment of the plasma cell malignancy, myeloma. However, it can also deplete benign plasma cells with the potential to reduce TRAb and alter the natural history of severe Graves' disease. This study aims to establish proof of concept that daratumumab has efficacy in severe Graves' disease patients and will provide important data to inform a choice of dosing regimen for subsequent trials.

Methods and Analysis: The Graves-PCD trial aims to determine if daratumumab modulates the humoral immune response in patients with severe Graves' disease, and if so, over what time period, and to find an optimal dose. It is a single blinded, randomised, dose-finding, adaptive trial using 4 different doses of daratumumab or placebo in 30 adult patients. Part 1 of the trial is dose-finding and, following an interim analysis; in part 2 the remaining patients will be randomised between the chosen dose(s) from the interim analysis or placebo. The primary outcome is the change in serum TRAb antibodies from baseline to 12 weeks.

Ethics and Dissemination

The trial received a favourable ethical opinion from London - Hampstead Research Ethics Committee (reference 21/LO/0449). The results of this trial will be disseminated at international meetings, in the peer-reviewed literature and through partner patient group newsletters and presentations at patient education events.

Registration

ISRCTN81162400

For peer review only

STRENGTHS AND LIMITATIONS OF THIS STUDY

- Current treatments for patients with severe Graves' disease are unsatisfactory and there is therefore significant unmet clinical need for new treatment
- Adaptive design allows efficient investigation of multiple doses
- The long-term remission of hyperthyroidism cannot be assessed in the 24 week follow-up period
- The duration of treatment or dosing intervals will not be tested in this study

For peer review only

INTRODUCTION

Graves' disease (autoimmune hyperthyroidism) affects around 3% of women and 0·5% of men, and most commonly presents in people in their 30s and 40s with a disproportionate burden of ill-health falling on working-age women.(1 ,2) Typical symptoms include weight loss, palpitations, heat intolerance, tremor, breathlessness, sweating, insomnia, loss of concentration and irritability. Around 40% of patients develop Thyroid Eye Disease (TED),(3) which can cause facial disfigurement as well as functional visual problems and even loss of sight. Quality of life in TED patients is poor, worse than for diabetes, and similar to patients with inflammatory bowel disease.(4) Rarely, Thyroid Dermopathy (Pretibial Myxoedema) may also occur leading to a brawny thickening of the skin on the lower legs and feet.(5) These latter 2 problems produce particularly distressing symptoms for a relatively young and active patient group. Around 10% of Graves' patients have a severe form of the disease, and experience severe thyrotoxicosis (serum FT4 ≥50pmol/l [upper limit of reference range 22pmol/l] or FT3≥15pmol [upper limit of reference range 6.8pmol/L]), failure of medical control of hyperthyroidism, large goitre, TED of sufficient severity to affect visual function or Thyroid Dermopathy. Current treatments for these patients with severe Graves' disease give unsatisfactory outcomes and are expensive, typically involving surgical thyroidectomy with or without several episodes of eye surgery.(6)

Conventional treatment of Graves' disease with antithyroid drugs (carbimazole, methimazole or propylthiouracil) leads to remission for only around 50% of patients, decreasing to less than 20% for patients with severe disease, and does not improve eye symptoms.(7 ,8) Other

For peer review only - http://bmjopen.bmj.com/site/about/guidelines.xhtml

treatment options of radio-active iodine or surgical thyroidectomy result in life-long hypothyroidism requiring levothyroxine therapy, which patients are often unwilling to accept.[9] Better treatment options are sorely needed for severe Graves' disease.

Severe Graves' disease is caused by high titres of directly pathogenic thyroid stimulating hormone receptor stimulating antibodies (TRAb), which are secreted from terminally differentiated B lymphocytes known as plasma cells.[10] Not only do TRAbs directly stimulate thyroid hormone over-production (leading to hyperthyroidism), they also cause thyrocyte hyperplasia leading to goitre, and bind to TSH receptors on the surface of orbital fibroblasts leading to mitogenic signalling that triggers and perpetuates eye disease. Failure of medical control and/or early relapse following conventional antithyroid drugs reflects the persistence of these long-lived, TRAb-secreting plasma cells in the secondary lymphoid tissues and bone marrow.

Both benign and malignant plasma cells express high levels of the cell-surface glycoprotein CD38. Daratumumab, a biological drug that targets CD38, was recently licenced for the plasma cell malignancy, myeloma.(9 ,10) This human anti-CD38 monoclonal antibody (mAb) has the potential to deplete plasma cells and therefore produce a rapid reduction in TRAb levels, which may alter the natural history of severe Graves' disease. This study aims to establish proof of concept that daratumumab has efficacy in severe Graves' disease patients and will provide important data to inform a choice of dosing regimen.

Tolbert et al. first reported the use of daratumumab in the treatment of benign, antibody-mediated haematological autoimmune disorders in 2016.(11) In this case a female in her late teens with autoimmune haemolytic anaemia (post stem cell transplant) was successfully treated with daratumumab and had an instant improvement in haemolysis. There are now numerous additional reports of daratumumab being repurposed to target benign disorders.[14]

Daratumumab is licenced for use in myeloma at a dose of 16mg/kg, however, patients with Graves' disease have several orders of magnitude fewer plasma cells and it is therefore expected that lower doses of drug will be active in this patient group with benign disease. To address this question the first stage of the study will be dose-finding. Notably, dose-finding trials in myeloma patients showed no difference in adverse event rates between doses of

For peer review only - http://bmjopen.bmj.com/site/about/guidelines.xhtml

8mg/kg and 16mg/kg, and no dose-limiting toxicity up to 24mg/kg.[11] Therefore, in order to define a signal for efficacy, this study will use 9mg/kg as the top dose in stage 1, with reducing concentrations (3mg/kg, 1mg/kg and 0.5mg/kg) along with placebo to determine the dose response in stage 1. As myeloma trials did not find increased adverse events or toxicities at higher doses, these four dose allocations will take place in parallel. Stage 2 of the study will randomise additional patients to one or more optimal doses of daratumumab, as selected from stage 1, or placebo to gain further information about real-life effect size, safety and tolerability.

METHODS AND ANALYSIS

This study protocol is based on the Standard Protocol Items: Recommendations for Interventional Trials (SPIRIT) 2013 statement.[15]

Sponsor and Trial Management

The trial Sponsor is the Newcastle upon Tyne Hospitals NHS Foundation Trust (Email: tnu-tr.sponsormanagement@nhs.net). Trial management is delegated to Newcastle Clinical Trials Unit, Newcastle University.

Objectives

The primary objective is to determine if daratumumab modulates the humoral immune response in patients with severe Graves' disease, with the primary outcome measure the change in serum TRAb antibodies from baseline to 12 weeks. Secondary objectives include determining the optimal dose of daratumumab, assessing safety, patient quality of life and impact on eye symptoms, thyroid function and goitre size (see Table 1).

Table 1: Objectives and Outcome Measures

	Objectives	Outcome Measures
**Primary**	To determine if daratumumab modulates the humoral immune response in Graves' disease patients	Change in serum TRAb antibodies from baseline to 12 weeks compared to change in placebo group
**Secondary**	To determine how fast daratumumab modulates the humoral immune response in Graves' disease patients	Change in serum TRAb antibodies from baseline to 2, 4, 6, 12 and 24 weeks

For peer review only

	To determine the optimal dose (or dose range) of daratumumab for Graves' disease patients	Dose-response curve for daratumumab against change in serum TRAb antibodies from baseline to 6 and 12 weeks
	To determine if daratumumab reduces thyroid hormone levels	Change in serum FT3 and FT4 from baseline to 2, 4, 6, 12 and 24 weeks
	To determine if daratumumab changes the time course of serum TSH	Change in serum TSH from baseline to 2, 4, 6, 12 and 24 weeks
	To determine if daratumumab changes thyroid size	Change in thyroid volume from baseline to 24 weeks measured by ultrasound
	To determine if daratumumab changes other thyroid autoantibodies	Change in serum ATPO and thyroglobulin antibodies from baseline to 6, 12 and 24 weeks
	To determine if daratumumab improves thyroid eye disease	Change in CAS, composite eye index and GOQoL score from baseline to 6, 12 and 24 weeks
	To determine if daratumumab improves thyroid symptom-related QoL	Change in ThyPRO39 score from baseline to 6, 12 and 24 weeks
	To determine if daratumumab is safe in this patient group	Change in serum immunoglobulins, specific antibodies including (SARS-CoV2) and blood count parameters from baseline to 6, 12 and 24 weeks.<br>Adverse Reactions to 24 weeks
**Experimental**	To determine if daratumumab changes lymphocyte/plasma cell transcriptomic markers	Analysis of blood plasma cell markers and mRNA signature
	To determine if daratumumab changes the lymphocyte subsets	Change in lymphocyte subsets (by FACS) from baseline to 6, 12 and 24 weeks

Footnotes: TRAb- TSH receptor antibodies; CAS- clinical activity score; GOQoL- Graves' orbitopathy quality of life instrument; ThyPRO39- thyroid patient reported outcome; FACS- fluorescent-activated cell sorting.

Trial Design

This is an adaptive, 2-stage randomised phase IIa, single centre clinical trial that will aim to recruit 30 patients with severe Graves' disease from secondary care at the Newcastle upon Tyne Hospitals NHS Foundation Trust. It is a single blinded trial in which participants will be blind to allocation. Recruitment start date: October 2021; planned end of trial (last patient last visit) April 2024.

Stage 1 is a dose-response study using 4 doses of daratumumab (9mg/kg, 3mg/kg, 1mg/kg, 0.5mg/kg) and a colourless, volume-matched placebo infusion in approximately 15 patients

For peer review only

For peer review only - http://bmjopen.bmj.com/site/about/guidelines.xhtml

(i.e. five groups of n=3, randomised in a 1:1:1:1:1 ratio using permuted random blocks without stratification) Figure 1.

Following Stage 1, an interim analysis will be performed of the first 15 patients in order to select an optimal dose(s) of daratumumab for Stage 2. The dose selection will be based on an analysis of the reduction in TRAb antibody concentration and safety assessed at 12 weeks. In stage 2, the remaining 15 patients will be randomised between placebo and one or two chosen doses of daratumumab depending on results of the interim analysis. Patients in both stages of the study will have all the same assessments and will be followed up for 24 weeks, with the primary endpoint being measured at week 12.

Figure 1: Design of study

Inclusion Criteria

1. Patients ≥18yrs old
2. Recent-onset Graves' disease (within 12 months) (defined as date of first thyroid function test showing hyperthyroidism (FT4 and TSH) in current episode).
3. TRAb antibody concentrations above 10U/L (on Roche or Brahms TRAb binding assays)
4. One or more of:
    - Pre-treatment severe hyperthyroidism (FT4 ≥50 pmol/L; or FT3 ≥15 pmol/l)
    - Persisting hyperthyroidism despite more than 12 weeks of antithyroid drug therapy (defined as FT3 above the upper limit of the reference range following 12 weeks of carbimazole treatment at a dose of 40mg or more daily (or equivalent dose of PTU))
    - Inflammatory thyroid eye disease (defined as clinical activity score, CAS≥3), or thyroid dermopathy
    - Large (visible) goitre (WHO grade III)
    - 2 or more relapses (3 episodes in total) despite completing 12 months or more of medical treatment on each occasion. Relapse is defined as FT3 above the upper limit of the local reference range.
5. For women of child-bearing potential, willing to use a highly effective contraceptive method during their participation in the trial.

For peer review only - http://bmjopen.bmj.com/site/about/guidelines.xhtml

6. Able to understand and speak sufficient English to complete trial procedures
7. Willing and able to provide informed consent prior to any trial procedures taking place

Exclusion Criteria

1. Previous thyroidectomy, or radioiodine treatment within 2 years
2. Pregnant or breastfeeding, or with a plan for pregnancy within 6 months
3. Previous shingles, known untreated cervical dysplasia, hepatitis B & C or HIV infection
4. Anaemia (Hb ≤ 100g/L), thrombocytopenia (≤75 x10^9/L) or neutropenia (≤1.0 x10^9/L)
5. Known chronic obstructive pulmonary disease (COPD) (defined as a forced expiratory volume [FEV] in 1 second <60% of predicted normal), persistent asthma, or a history of asthma within the last 2 years (intermittent asthma without hospitalisation is allowed)
6. Any significant physical or mental health condition that impacts the safety of the intervention, the interpretation of thyroid function or the ability of a participant to attend for intervention and safety monitoring, eg. major cardiorespiratory disease, renal or hepatic failure, pancreatitis, cancer undergoing active treatment (excluding non-melanoma skin cancer), untreated chronic infection including TB, psychosis, depression impairing Activities of Daily Living
7. Current use of immunosuppressive therapy for thyroid eye disease or other conditions (within 3 months)
8. Current or previous participation in a CTIMP research study within 4 months
9. Hypersensitivity or anaphylactic reaction to previous monoclonal antibody treatments or methylprednisolone
10. Inability, in the opinion of the investigator, to be able to complete the clinical trial visits or procedures.

Risk Assessment

Daratumumab is a licenced product for myeloma patients and the main risk is that it will have a different side effect profile in patients with benign disease. The typical risks of daratumumab when used for myeloma include transient infusion reactions and haematological dyscrasias, the latter being accounted for by the bone marrow suppression in myeloma patients and leading to a small risk of infection (≤5%).

For peer review only - http://bmjopen.bmj.com/site/about/guidelines.xhtml
For peer review only

Daratumumab interferes with urgent blood cross matching and all patients will have a Coombs test and blood group and transfusion antibody screen prior to administration of daratumumab, as well as receiving an information card in case blood transfusion is needed. There is a potential for reactivation of latent viral illness, and so all participants will be screened for hepatitis B, C and HIV infection, and participants with previous shingles or current untreated cervical dysplasia will be excluded. There are no specific risks demonstrated in the Graves' disease population who will be younger and in better overall health than the patient group who currently receive daratumumab for myeloma.

Participants will be offered a course of SARS-CoV2 vaccination prior to treatment with IMP. If loss of immunity to specific pathogens is detected at the end of the study, the participants may be offered repeat vaccination for tetanus, pneumococcus and SARS-CoV2, where clinically appropriate. Throughout their participation in the study, patients will continue their regular "background" medication for treatment of thyrotoxicosis (i.e. antithyroid drugs, with or without levothyroxine) as prescribed by their clinician as per usual clinical care and will be carefully monitored with regular blood tests throughout the study.

Patient Identification, Consent and Screening

Potential participants with severe Graves' disease within secondary care will be identified and approached in person, by post or email with a trial participant information sheet (PIS) by one of the clinical team responsible for their usual management. Potential participants will also be identified and referred by local secondary care Patient Identification Centre sites, or can self-refer to the trial via adverts through organisations such as the British Thyroid Foundation (BTF). Consent discussions and written informed consent will take place in person at a research specific clinic appointment, with the principal investigator (PI) or alternative member of the research team who is medically qualified and appropriately trained. For women of child-bearing potential, consent discussions will include the need to use a highly effective form of contraception (as listed on the PIS) for the duration of their participation in the trial. Consent discussions will be documented in medical notes.

Following written informed consent, eligibility for the trial will be assessed by:

- Demographics and medical history including medication history

For peer review only - http://bmjopen.bmj.com/site/about/guidelines.xhtml

- Full external medical examination, including weight, heart (including resting pulse rate and 12 lead ECG), chest, abdomen, goitre size assessment, skin, eyes and neurological examination
- Blood tests (FBC, U&E, LFT, HBsAg, HCV antibodies, HIV, Bone chemistry, TRAb antibodies, serum FT4, FT3, TSH, serum IgGAM, Coombs, blood group and red cell phenotyping)
- A urine sample pregnancy test for all female participants of child-bearing potential (to be repeated where there is >14 days between screening and baseline visits)

The trial screening log will include reasons for screen fails and any reasons given for declining the trial.

Randomisation

Randomisation will be performed by delegated and trained members of the site research team using the online system "Sealed Envelope"; a central, secure, 24-hour web-based randomisation system. In both stages, the allocation sequence will be computer-generated using a random permuted block design. Patients will be randomly allocated equally to the available daratumumab doses or to a matched placebo; for example, if 2 doses of daratumumab plus placebo are to be used, the allocation would be 1:1:1. There are no stratification factors.

In Stage 1, patients will be allocated to receive a dose of daratumumab (9, 3, 1, 0.5mg/Kg) or matched placebo. In Stage 2, patients will be allocated to receive daratumumab (one or two dose levels as determined by the interim analysis), or matched placebo.

Intervention

Each participant will receive 2 infusions, on day 0 and day 14 (+/-3 days). The prescribed daratumumab dose will be prepared in a 500 or 1000ml bag of sodium chloride 0.9%, using the most recent available participant weight to calculate banded dose (see Table 2 for volume and infusion rate). Each participant will be prescribed the same banded dose for both infusions, using the same weight for both. Where placebo is allocated, a volume-matched bag of sodium chloride 0.9% will be used. All participants will be pre-medicated with three non-investigational medicinal products (NIMPs), paracetamol (1g po), methylprednisolone (100mg IV) and chlorphenamine (10mg IV) at least 30 minutes prior to their allocated infusion.

For peer review only - http://bmjopen.bmj.com/site/about/guidelines.xhtml

Table 2: Infusion Rate Table

Time (Hr:Min)	Rate (mls /Hr)	Total volume at end of time (ml)
**Stage 1 (1000mls)**		
1	80	80
2	160	240
3	240	480
4	320	800
4:40	320	1000
**Stage 2 rate A (1000mls)**		
1	50	50
2	100	150
3	150	300
4	200	500
5	200	700
6	200	900
6:30	200	1000
**Stage 2 rate B (500mls)**		
1	50	50
2	100	150
3	150	300
4	200	500

Table 2: The maximum infusion rate of daratumumab was determined by the recommendation of the SmPC for daratumamab (DARZALEX 20 mg/mL concentrate for solution for infusion). For Stage 1, all participants will receive the IMP/placebo diluted in 1000mls sodium chloride 0.9% for both infusions. For stage 2, some patients may receive higher doses of daratumumab, so a more prolonged first infusion will be given to all participants. If participants have no IRRs during their first dose in stage 2, then the second dose may be given in 500mls sodium chloride 0.9% with a faster infusion rate, in line with SmPC recommendations.

Vital signs (pulse rate, blood pressure and temperature) will be recorded prior to the start of each infusion and every 15 minutes during the course of the infusion, and for 1 hour afterwards. Infusion related reactions will be addressed as shown in Table 3, and documented as an adverse event. Infusion charts will be completed to document compliance with prescribed dose. Participants will be followed up by phone approximately 2 days after each infusion visit to check for any adverse events. After the day 14 visit, no further IMP will be administered. Patients will continue their regular medications, including antithyroid drugs and betablockers throughout the study, as deemed appropriate by the clinical team.

For peer review only - http://bmjopen.bmj.com/site/about/guidelines.xhtml

Table 3: Infusion Related Reactions

**Grade 4 reactions**	Life-threatening	Stop and permanently discontinue infusion.
**Grade 3 reactions**	Severe, including:<br>• Tachycardia ≥120BPM or drop in SBP ≥30mmHg over pre-infusion with symptoms<br>• Rise in SBP ≥30mmHg over pre-infusion or tachypnoea ≥24/min<br>• Hypoxia SaO2 <92%	Stop infusion until observations improve and restart at 50% of the infusion rate at which IRR occurred. If the patient does not experience additional symptoms, infusion rate escalation may be resumed at increments and intervals as appropriate. The procedure above should be repeated in the event of recurrence of Grade 3 symptoms. After the infusion re-start attempts, the IMP treatment will be permanently discontinued upon the third occurrence of a Grade 3 or greater IRR.
**Grade 1-2 reactions**	Mild to moderate, including:<br>• nasal congestion<br>• cough<br>• chills<br>• throat irritation<br>• vomiting<br>• nausea	Stop infusion in case of clinically significant reaction only until observations improve and restart at 50% of the infusion rate at which IRR occurred. If the patient does not experience any further IRR symptoms, infusion rate escalation may be resumed at increments and intervals as clinically appropriate up to the maximum rate. Infusion does not need to be stopped if IRR is not clinically significant.

For peer review only

Assessments

Follow up visits will take place at weeks 4, 6, 12 and 24 (+/- 1 week); the details of assessments are provided in Table 4. At each visit, concomitant medications will be recorded, an adverse event check will be performed, compliance with anti-thyroid medication checked and full blood count and thyroid function (TSH, FT4, FT3 and TRAb) will be assessed. Other assessments include goitre size assessment, eye exam, patient reported quality of life measures (GOQoL(4) and ThyPRO[16]), specific antibody tests and other safety bloods (FBC, LFT). At the end of their participation in the trial, participants will be asked their opinion on which treatment allocation they received, and if they would recommend their treatment to a friend with the same condition. In addition, we plan to undertake exploratory analyses of lymphocyte subsets determined by flow cytometry and transcriptomic markers of plasma cells using qRT-PCR.[17]

For peer review only - http://bmjopen.bmj.com/site/about/guidelines.xhtml

Table 4: Schedule of Assessments

Assessment/Activity	Visit 1	Visits 2 & 3	Visit 4	Visit 5	Visit 6	Visit 7	Visit 8	Visit 9
			Day 0	Day 14 (+/-3 days)	4 weeks (+/- 1 week)	6 weeks (+/- 1 week)	12 weeks (+/- 1 week)	24 weeks (+/- 1 week)
Written Informed Consent	X							
Demographics & medical history	X							
Physical exam	X							
12-lead ECG	X							
Eye exam (CAS)	X							
Hep B & C serology, HIV	X							
Bone chem	X							
Coombs, blood group, RBC phenotyping	X							
Pregnancy testing (females)	X						X	
COVID vaccination offered		X X						
IMP infusion			X	X				
Weight	X	X	X	X	X	X	X	X
Resting pulse rate	X		X	X	X	X	X	X
Goitre size assessment	X		X			X	X	X
FBC	X		X	X	X	X	X	X
TSH, FT4, FT3, TRAb	X		X	X	X	X	X	X
U&E, LFT	X		X	X	X	X		
IgGAM	X		X			X	X	X
Height			X					
Eye exam (composite eye index)			X			X	X	X
Thyroid volume USS			X					X
QoL questionnaires (GOQoL, ThyPRO)			X			X	X	X
TgAb, ATPO			X			X	X	X
Specific antibodies			X				X	X
Exploratory analysis sample			X			X	X	X
Vital signs			X	X				
Compliance with ATD			X	X	X	X	X	X
Concomitant medication		X	X	X	X	X	X	X
Adverse event reporting		X	X	X	X	X	X	X
Follow-up phone call			X	X				
End of trial questions								X

Discontinuation of Treatment and Withdrawal

Participants who have completed one or more daratumumab/placebo infusions may remain on the trial but choose to discontinue treatment. These participants will continue to take part in the trial assessments as per the trial schedule of events. Alternatively, an investigator may discontinue a participant from receiving trial treatment at any time (until administration of

For peer review only

second infusion is completed), if the investigator considers it necessary for any reason. Participants who withdraw from the trial after receiving IMP (full or part dose) will not be replaced. Participants who withdraw before IMP is given will be replaced.

Safety Reporting

Adverse events (AEs) will be documented in patient medical notes -before entry into the trial database. AEs judged by the investigator responsible for patient care as consistent with the usual clinical pattern for patients with severe Graves' disease (e.g. Hyperthyroidism, hypothyroidism, tachycardia, bradycardia) will not be reported. Disease progression will be recorded as an AE only if it is more severe than expected in the trial population. AEs will be coded according to MedDRA classifications. All AEs meeting the criteria for seriousness (SAEs) will be reported by the investigator to the trial Sponsor within 24 hours of awareness, and suspected unexpected serious adverse reactions will be reported to the MHRA and research ethics committee (REC) by the trial Sponsor in line with regulatory reporting timelines.

End of Trial

The end of the trial is defined as last patient last visit (LPLV). Treatment allocation will be revealed to all participants (and their GP/referring clinician where applicable) by letter at the end of the trial, with contact details to discuss if required. At the end of their participation in the study participants will return to standard NHS care.

Payment

No payment will be made to participants for taking part in this trial, although reasonable travel expense reimbursement including taxis will be made for trial visits.

Data Management

The Clinical Data Management System (CDMS) used to capture trial data is Red Pill, supplied by Sealed Envelope. Users have individual password protected user accounts appropriate to their trial duties (e.g. randomisation, data entry, monitor). Patients are identified on the CDMS using a unique participant ID. Data monitoring will include a mixture of central, off-site and on-site approaches and will be outlined in a risk-based monitoring plan prior to the start of the trial. The trial specific Data Management Plan will include details of how the validity

For peer review only - http://bmjopen.bmj.com/site/about/guidelines.xhtml

and quality of data will be monitored and managed. As far as possible, validations will be built into the CDMS and additional manual validations will be detailed in the Data Validation Plan. Trial monitoring will be performed by NCTU as per the monitoring plan and the trial is also liable to audit by the trial Sponsor and regulator.

Until publication of the trial results, access to the full dataset will be restricted to the Trial Management Group (TMG) and to authors of the publication. Requests for anonymised data should be directed to the lead author.

Analysis

The primary analysis will assess whether there is a significant dose-response using percentage reduction in serum TRAb concentration from baseline to 12 weeks using the intention-to-treat (ITT) population (participants analysed according to randomisation allocation).

To do this we will calculate a contrast-based test statistic at a one-sided 5% type I error rate.[18] If this test statistic is significant, we will test whether there is a mean difference in the outcome between individual doses and placebo using a suitable linear model. We will also perform a simple test of all placebo participants vs all daratumumab patients using a linear model that adjusts for presence of eye disease. We will also fit quadratic and three-parameter Emax models to assess dose-response.[19] This analysis will inform selection of the most suitable dose for subsequent trials. Exploratory analyses using subgroups of patients including by gender, smoking status, presence of TED and change in serum TRAb will be undertaken.

Interim Analyses and Criteria for the Premature Termination of the Trial

An interim analysis of dose-response will be conducted after 15 patients have provided 12-week follow-up data. To assess efficacy, a three-parameter Emax model will be fitted to the 12-week TRAb log-percentage change using the DoseFinding package in R(12 ,13) in the dose-response population (participants analysed according to the amount of daratumumab received). This model is used to estimate the plateau effect and i) the dose that gives a mean reduction of 50% and ii) the dose that gives 90% of the plateau effect. If the estimated plateau effect corresponds to >50% reduction in TRAb, the trial will continue with placebo and doses (i) and (ii) defined above. If the estimated plateau corresponds to less than 25% reduction in TRAb, the trial will terminate early for lack of promising dose-response unless the DMC agree that there has been an unequivocal improvement in serum thyroid hormone measurements

For peer review only

in the active IMP vs placebo groups; if it is between 25%-50% reduction, the trial will continue with doses of 9mg/kg and a higher daratumumab dose (e.g. 12 or 16mg/Kg as determined by safety data), plus placebo. Safety information will be taken into consideration in deciding stage 2 dose(s) and could over-ride the model-based dose recommendations.

Statistical Size Calculations

A 50% or more reduction in TRAb antibody concentration from baseline within 12 weeks of intervention was chosen as biologically plausible and clinically important. An observational study of milder Graves' disease patients showed a mean change in TRAb concentration of 2% over 6 weeks, with an SD of 15%. Assuming: a standard deviation of 25%, to account for the outcome being measured at 12 weeks; a 5% reduction in placebo treated subjects; an Emax dose-response; a plateau 60% reduction in TRAb concentration over 12 weeks with active IMP; and 1mg/kg dose giving half of the total, 27 participants will give over 90% power (5% one-sided type I error) to conclude there is a dose response relationship. In order to allow for 10% drop out, we will plan to recruit 30 participants.

Power was verified through simulations in 'R',(13) assuming the logarithm of the percentage reduction to be normally distributed with SD of 0.5 (equivalent to 25% SD when the mean is 50% reduction). The power/type I error rate for a range of scenarios is given in Table 5.

Table 5: Power/type 1 error rate calculations

**Scenario**	**Probability to conclude dose-response (10000 replicates)**
1 – plateau of 80% reduction ED50 =3mg/kg	>99%
2 – plateau of 60% reduction, ED50=1mg/kg	92%
3 – plateau of 60% reduction, ED50=3mg/kg	83%
4 – null scenario	3.2%

Footnote: The R code required to reproduce these results can be found in supplementary material 1.

Trial Oversight

The TMG will meet approximately monthly throughout the trial, and includes the CI, statisticians, Newcastle Clinical Trials Unit staff, pharmacy and Sponsor representatives. Oversight is provided by an independent Data Monitoring Committee who will meet at the start of the trial, at the interim analysis point and on an ad hoc basis throughout, as well as a

For peer review only - http://bmjopen.bmj.com/site/about/guidelines.xhtml

Trial Steering Committee (TSC) who will generally meet following a DMC meeting. The terms of reference and composition of these committees are outlined in their respective charters.

Protocol Amendments

NCTU will submit amendments to the HRA and MHRA/REC (as appropriate) as well as informing ISRCTN, trial recruiters, investigators and the relevant R&D department of changes. A list of version changes will be kept in the trial protocol appendix and can be found in supplementary material 2.

Trial Status

This manuscript is based on protocol version 6.0 dated 18 May 2023. The Graves-PCD trial opened to recruitment on 29 September 2021. At the time of writing, stage 2 of the trial is underway.

Patient and Public Involvement

Patients were involved in the design of the trial via the British Thyroid Foundation (www.btf-thyroid.org), and lay members will have oversight and input during the trial and dissemination as members of the TSC.

ETHICS AND DISSEMINATION

The trial received a favourable ethical opinion from London - Hampstead Research Ethics Committee (reference 21/LO/0449). The results of this trial will be disseminated at international meetings, in the peer-reviewed literature and through partner patient group newsletters and presentations at patient education events.

ACKNOWLEDGEMENTS

We would like to acknowledge the ongoing support provided by the British Thyroid Foundation. We are grateful to Prof Ruth Andrew of Edinburgh University for advice concerning dose choices and to Dr. Catherine Stroud of Newcastle Hospitals NHS Trust for immunological safety advice.

AUTHORS' CONTRIBUTIONS

For peer review only

SHP had the original idea for the trial. The trial protocol was developed by SHP, with substantial input and revision by FW, JWas, MC, RM, JWal, LG and PB. IB and NM also made changes to the protocol at various stages of development. The trial protocol manuscript was primarily drafted by FW, with substantial input from SHP and JWas. All other coauthors approved the final version of the manuscript.

COMPETING INTERESTS STATEMENT

SHP has received speaker fees from Merck, IBSA, and consulted for Apitope/Worg and Immunovant/Roivant. JWas has consulted for Worg. No other author has conflicts of interest to declare.

FUNDING STATEMENT

This trial is funded by the UKRI Medical Research Council (MRC), ref MR/V005898/1

REFERENCES

1. Nyström HF, Jansson S, Berg G. Incidence rate and clinical features of hyperthyroidism in a long-term iodine sufficient area of Sweden (Gothenburg) 2003-2005. *Clin Endocrinol (Oxf)* 2013;**78**(5):768-76.
2. Vanderpump MP. The epidemiology of thyroid disease. *Br Med Bull* 2011;**99**:39-51.
3. Chin YH, Ng CH, Lee MH, et al. Prevalence of thyroid eye disease in Graves' disease: A meta-analysis and systematic review. *Clinical Endocrinology* 2020;**93**(4):363-74.
4. Gerding MN, Terwee CB, Dekker FW, et al. Quality of life in patients with Graves' ophthalmopathy is markedly decreased: measurement by the medical outcomes study instrument. *Thyroid* 1997;**7**(6):885-9.
5. Fatourechi V, Pajouhi M, Fransway AF. Dermopathy of Graves disease (pretibial myxedema). Review of 150 cases. *Medicine (Baltimore)* 1994;**73**(1):1-7.
6. Estcourt S, Hickey J, Perros P, et al. The patient experience of services for thyroid eye disease in the United Kingdom: results of a nationwide survey. *Eur J Endocrinol* 2009;**161**(3):483-7.
7. Struja T, Fehlberg H, Kutz A, et al. Can we predict relapse in Graves' disease? Results from a systematic review and meta-analysis. *Eur J Endocrinol* 2017;**176**(1):87-97.
8. Eckstein AK, Plicht M, Lax H, et al. Thyrotropin receptor autoantibodies are independent risk factors for Graves' ophthalmopathy and help to predict severity and outcome of the disease. *J Clin Endocrinol Metab* 2006;**91**(9):3464-70.
9. Lokhorst HM, Plesner T, Laubach JP, et al. Targeting CD38 with Daratumumab Monotherapy in Multiple Myeloma. *New England Journal of Medicine* 2015;**373**(13):1207-19.
10. Lonial S, Weiss BM, Usmani SZ, et al. Daratumumab monotherapy in patients with treatment-refractory multiple myeloma (SIRIUS): an open-label, randomised, phase 2 trial. *Lancet* 2016;**387**(10027):1551-60.
11. Tolbert VP, Goldsby R, Huang J, et al. Daratumumab Is Effective in the Treatment of Refractory Post-Transplant Autoimmune Hemolytic Anemia: A Pediatric Case Report. *Blood* 2016;**128**(22):4819-19.

For peer review only

12. Pinheiro J, Bornkamp B, Glimm E, et al. Model-based dose finding under model uncertainty using general parametric models. *Statistics in Medicine* 2014;**33**(10):1646-61.
13. R Core Team. R: A Language and Environment for Statistical Computing. Vienna, Austria: R Foundation for Statistical Computing, 2024:https://www.R-project.org/.

For peer review only

For peer review only - http://bmjopen.bmj.com/site/about/guidelines.xhtml

Figure 1: Design of study

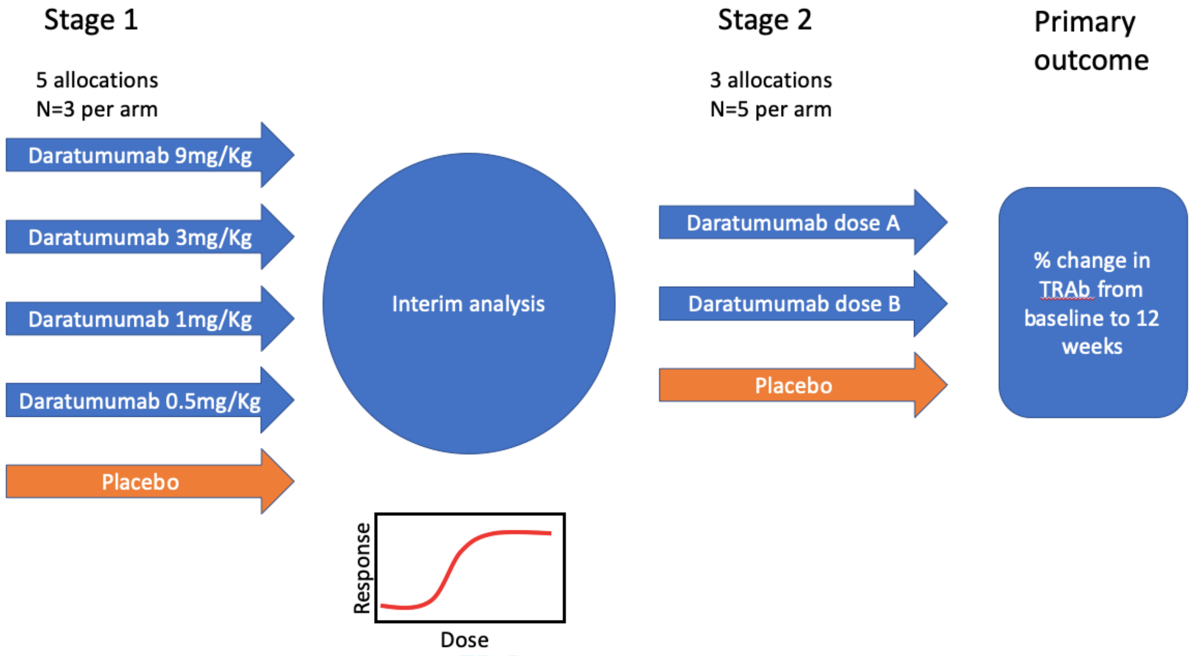

For peer review only - http://bmjopen.bmj.com/site/about/guidelines.xhtml

```
library(boot)
library(MCPMod)
library(DoseFinding)


#Assume that -log(percentage of baseline) (-log of ratio of follow-up TRAb to baseline TRAb) is normally distributed and the outcome


#Function to run a given number of simulation replicates:

simulation=function(
  initialdoses=c(0,0.5,1,3,9), #Initial range of doses given
  stage1.nperdose=3, #Initial number of patients per dose in stage 1
  maximumpercentagereduction=80, #plateau percentage reduction from baseline
  minimumpercentagereduction=5,#mean reduction with placebo
  ed50=2,#dose that gives 50% of effect
  errSD=sqrt(1/4),#SD of transformed outcome (assuming the SD is 25 at the mean of 50% reduction) is approximately sqrt(1/50^2 * 25^2)
  alpha=0.05, # the alpha level
nreplicates=1000 #number of simulation replicates to run
)
{
  #define vectors to track statistical properties

  power.contrast=rep(0,nreplicates)
estimated.dose50=rep(NA,nreplicates)
estimated.ed50=rep(NA,nreplicates)

selectedstage2doses=matrix(NA,nreplicates,3)
xdose<-initialdoses
nsam<-stage1.nperdose #number of patient per dose

#Hypothesised dose response

#transform of maximum %age reduction
ehi<- -log((100-maximumpercentagereduction)/100)
#Transform for placebo reduction
elo<- -log((100-minimumpercentagereduction)/100)


for(replicate in 1:nreplicates)
{

#sim first stage data
dfData.stage1 <- genDFdata("emax", c(e0 = elo, eMax = ehi-elo, ed50 = ed50), doses=xdose, sigma=errSD, n=nsam)


#plot(dfData.stage1$dose,dfData.stage1$resp)
#points(aggregate(dfData.stage1$resp, list(dose=dfData.stage1$dose), mean),pch=15)

#fit an ANOVA model to estimate means
fit.aovmean = lm(resp~as.factor(dose)-1, data=dfData.stage1) #no intercept
#summary(fit.aovmean)
EstMean.aov = coef(fit.aovmean) # Extract the estimated sample means
aov.sigma = summary(fit.aovmean)$sigma # Extract the estimated sigma

# PoC: contrast mean >0 statistically?
len.Dosage<-length(xdose)

n=nsam # sample size for each dosage
# The c's for 4-dose and 1-placebo
Contrast.Coef = c(-2,-1,0,1,2)
# Implementation of equation 9.2. with 1-sided test "Dose Finding in Drug Development" Naitee Ting. Springer
# where "qt" is for the quantile-t with one-sided (i.e. 1-alpha)

teststatistic.contrast=sum(Contrast.Coef*EstMean.aov)/(aov.sigma*sqrt(sum(Contrast.Coef^2/n)))
pvalue=1-pt(teststatistic.contrast,df=(n-1)*len.Dosage)
PoC.Contrast=sum(Contrast.Coef*EstMean.aov)/(aov.sigma*sqrt(sum(Contrast.Coef^2/n))) >qt(1-alpha,df=(n-1)*len.Dosage)

# fit the "Emax" model
fitemax = fitMod(dose, resp, data=dfData.stage1, model="emax",  bnds = c(0.01, 25))
# Call "plot" to plot the Emax fit
#plot(fitemax,CI=TRUE,plotData="meansCI",xlab="Dose",ylab="Response")


#function to find dose achieving reponse of 50% and bootstrap CI



#If e0+eMax is less than the equivalent of a 25% reduction, then stop early:

if((fitemax$coefs[1]+fitemax$coefs[2])< (-log(0.75)))
{
  next;
}

#If e0+eMax is between 25% and 50% reduction, then second stage doses should be 0, 3 and 9:

if((fitemax$coefs[1]+fitemax$coefs[2])< (-log(0.5)))
{

  xdose.stage2.overrun<-c(0,0.5,1,3,9)
  dfData.stage2.overrun <- genDFdata("emax", c(e0 = elo, eMax = ehi, ed50 = ed50), doses=xdose.stage2.overrun, sigma=errSD, n=1)
  xdose.stage2.afterinterim=c(0,3,9)

  selectedstage2doses[replicate,]=c(0,3,9)
```

```
}

#If e0+eMax is higher than 50% reduction, then select dose that gives 50% reduction and one that gives 90% of plateau reduction
if((fitemax$coefs[1]+fitemax$coefs[2])>= (-log(0.5)))
{

#extra dose chosen to be close to plateau effect:
plateau=fitemax$coefs[1]+fitemax$coefs[2]

#find dose that gives 90% of plateau effect:

dose.nearplateau=doseforgivenmean(fitemax,mu=0.9*plateau)

dose.nearplateau=round(dose.nearplateau)



#Estimated dose that gives 50% reduction, or 50% of plateau if plateau is lower than 50:

dose.50=doseforgivenmean(fitemax,mu=-log(0.5))
#round to nearest dose:

dose.50=round(dose.50)
#If rounded dose.50 is less than or equal to 0 (can be less if e0 is estimated to be high), set to 0.5:

if(dose.50<=0)
{
  dose.50=0.5
}

#If rounded dose.50 is >9, set to 6:

if(dose.50>9)
{
  dose.50=6
}

#If rounded dose.plateau is <= dose.50, set to dose.50+1:

if(dose.nearplateau<=dose.50)
{
  dose.nearplateau=dose.50+1
}

#If rounded dose.nearplateau is >9, set to 9:

if(dose.nearplateau>9)
{
  dose.nearplateau=9
}



#To allow for overrun, secondstage doses include 5 more recruited under the stage 1 allocation, then remaining allocated 3,3,3 to new
#Note this only makes 29

xdose.stage2.overrun<-c(0,0.5,1,3,9)
dfData.stage2.overrun <- genDFdata("emax", c(e0 = elo, eMax = ehi, ed50 = ed50), doses=xdose.stage2.overrun, sigma=errSD, n=1)
xdose.stage2.afterinterim<-c(0,dose.50,dose.nearplateau)

selectedstage2doses[replicate,]=c(0,dose.50,dose.nearplateau)

}

dfData.stage2.afterinterim <- genDFdata("emax", c(e0 = elo, eMax = ehi, ed50 = ed50), doses=xdose.stage2.afterinterim, sigma=errSD, n=

dfData.combined=rbind(dfData.stage1,dfData.stage2.overrun,dfData.stage2.afterinterim)


#Final analysis:

#fit an ANOVA model to estimate means
fit.aovmean = lm(resp~as.factor(dose)-1, data=dfData.combined) #no intercept
summary(fit.aovmean)
EstMean.aov = coef(fit.aovmean) # Extract the estimated sample means
aov.sigma = summary(fit.aovmean)$sigma # Extract the estimated sigma

#Set up contrast: check if even or odd number of unique doses

ndoses=length(unique(dfData.combined$dose))
if(ndoses==5){Contrast.Coef<-c(-2,-1,0,1,2)}
if(ndoses==6){Contrast.Coef<-c(-5,-3,1,1,3,5)} #notice change in contrast from -3,2,-1,1,2,3 so equal spacing throughout
if(ndoses==7){Contrast.Coef<-c(-3,-2,-1,0,1,2,3)}


n=as.double(table(sort(dfData.combined$dose))) # sample size for each dosage

# Implementation of equation 9.2. with 1-sided test "Dose Finding in Drug Development" Naitee Ting. Springer
# where "qt" is for the quantile-t with one-sided (i.e. 1-alpha)


teststatistic.contrast=sum(Contrast.Coef*EstMean.aov)/(aov.sigma*sqrt(sum(Contrast.Coef^2/n)))
```

```
pvalue=1-pt(teststatistic.contrast,df=2*ndoses)

power.contrast[replicate]=ifelse(pvalue<alpha,1,0)


fitemax = fitMod(dose, resp, data=dfData.combined, model="emax",  bnds = c(0.01, 25))

estimated.dose50[replicate]=doseforgivenmean(fitemax,-log(0.5))


estimated.ed50[replicate]=fitemax$coefs[3]


}

return(list(power.contrast=mean(power.contrast),conclude.doseeffect=power.contrast,estimated.dose50=estimated.dose50,estimated.ed50=es
}




#Function to find dose that gives target response assuming three-parameter emax
doseforgivenmean <- function(model, mu) {


  return((model$coefs[3]*(mu-model$coefs[1]))/(model$coefs[2]+model$coefs[1]-mu))
}


#Function for bootstrapping  #currently not modified to work with transformed outcome
dr50norm.boot <- function(data, indices) {
  y <- fit + e[indices]
  fitemaxboot = fitMod(data$dose, y, model="emax",  bnds = c(0.01, 50))
  est.parm = coef(fitemaxboot)
  ED50 = est.parm["ed50"] # extract the estimated ED50
  EMAX = est.parm["eMax"] # extract the estimated EMAX
  E0 = est.parm["e0"] # extract the estimated E0
  phi <- unname((50-E0)/EMAX)
  DoseR <- unname(phi*ED50/(1-phi))
  return(DoseR)
}

 #Scenario 1: large plateau effect, emax model
set.seed(1)
results.scenario1=simulation(nreplicates=10000,ed50 = 3)
print(c("Median ED50: ",median(results.scenario1$estimated.ed50,na.rm=T)))
print(c("Power: ",results.scenario1$power.contrast))

#Scenario 2: medium plateau effect, emax model
set.seed(1)
results.scenario2=simulation(maximumpercentagereduction = 60,ed50=1,nreplicates=10000 )
print(c("Median ED50: ",median(results.scenario2$estimated.ed50,na.rm=T)))
print(c("Power: ",results.scenario2$power.contrast))


#Scenario 3: medium plateau effect, emax model with ED50 equal to 3
set.seed(1)
results.scenario3=simulation(maximumpercentagereduction = 60,ed50=3,nreplicates=10000 )
print(c("Median ED50: ",median(results.scenario3$estimated.ed50,na.rm=T)))
print(c("Power: ",results.scenario3$power.contrast))


#Scenario 4: null:
set.seed(1)
results.scenario4=simulation(minimumpercentagereduction = 5,maximumpercentagereduction = 5,ed50=100,nreplicates=10000 )
print(c("Median ED50: ",median(results.scenario4$estimated.ed50,na.rm=T)))
print(c("Power: ",results.scenario4$power.contrast))
```

Graves-PCD: Protocol for a randomised, dose-finding, adaptive trial of the plasma cell depleting agent daratumumab in severe Graves' disease

Supplementary Material 2

Table S2: Protocol Version History

Protocol version no.	Details of changes made
6.0	4. Trial Design- update to Figure 2<br>5. Trial Settings update<br>6.1. Update to inclusion criteria 2 and 4<br>7.2.1. Patient Identification- Addition of self-referral participant information<br>8.6. Dosage Schedule & Modifications- Clarification on IRRs<br>Appendix 3- Update to Infusion Rate Tables text and Infusion Related Reactions (IRR) concerning Grade 1-2 reactions management.
5.0	8.6 update to Dosage Schedule & Modifications<br>Appendix 3- 16.3.2 update to Infusion Rate Tables text
4.0	Update to Key Trial Contacts<br>Appendix 3- Update to Dose Banding Tables, Infusion Rate Table and Infusion Related Reactions (IRR)
3.0	Update to Key Trial Contacts<br>Typographical corrections<br>7.1.8 Storage of TRAb sample until data lock at visits 4, 7, 8 and 9<br>7.1.11 Addition of end of trial questions<br>7.7 Removal of reconfirmation of eligibility >6 weeks, addition of re-screening >3 months
2.0	2. Addition of rationale for choice of doses in stage 1<br>6.2 Update to exclusion criterion 6<br>7.1.5 Addition of photography for patients with thyroid eye disease<br>7.12 Removal of use of initials<br>8.3 and 9.3.1 Removal of reference to SmPC section 4.5<br>9.4 Addition of yellow card reporting for NIMPs<br>10.1.4 Clarification on criteria for safety assessment at interim analysis<br>16.3.3 Update to response to IRRs
1.0	Original version

For peer review only - http://bmjopen.bmj.com/site/about/guidelines.xhtml

SPIRIT

STANDARD PROTOCOL ITEMS: RECOMMENDATIONS FOR INTERVENTIONAL TRIALS

SPIRIT 2013 Checklist: Recommended items to address in a clinical trial protocol and related documents*

Section/item	Item No	Description	Addressed on page number
**Administrative information**			
Title	1	Descriptive title identifying the study design, population, interventions, and, if applicable, trial acronym	__1_________
Trial registration	2a	Trial identifier and registry name. If not yet registered, name of intended registry	__2_________
	2b	All items from the World Health Organization Trial Registration Data Set	throughout
Protocol version	3	Date and version identifier	__17________
Funding	4	Sources and types of financial, material, and other support	__2_________
Roles and responsibilities	5a	Names, affiliations, and roles of protocol contributors	__1, 19______
	5b	Name and contact information for the trial sponsor	___5_________
	5c	Role of study sponsor and funders, if any, in study design; collection, management, analysis, and interpretation of data; writing of the report; and the decision to submit the report for publication, including whether they will have ultimate authority over any of these activities	__17________
	5d	Composition, roles, and responsibilities of the coordinating centre, steering committee, endpoint adjudication committee, data management team, and other individuals or groups overseeing the trial, if applicable (see Item 21a for data monitoring committee)	____17_______

For peer review only - http://bmjopen.bmj.com/site/about/guidelines.xhtml

**Introduction**			
Background and rationale	6a	Description of research question and justification for undertaking the trial, including summary of relevant studies (published and unpublished) examining benefits and harms for each intervention	____3-5______
	6b	Explanation for choice of comparators	_____4/5_____
Objectives	7	Specific objectives or hypotheses	_____5/6_____
Trial design	8	Description of trial design including type of trial (eg, parallel group, crossover, factorial, single group), allocation ratio, and framework (eg, superiority, equivalence, noninferiority, exploratory)	_____6______
**Methods: Participants, interventions, and outcomes**			
Study setting	9	Description of study settings (eg, community clinic, academic hospital) and list of countries where data will be collected. Reference to where list of study sites can be obtained	_____6_______
Eligibility criteria	10	Inclusion and exclusion criteria for participants. If applicable, eligibility criteria for study centres and individuals who will perform the interventions (eg, surgeons, psychotherapists)	_____7-9_____
Interventions	11a	Interventions for each group with sufficient detail to allow replication, including how and when they will be administered	_____11/12____
	11b	Criteria for discontinuing or modifying allocated interventions for a given trial participant (eg, drug dose change in response to harms, participant request, or improving/worsening disease)	_____12______
	11c	Strategies to improve adherence to intervention protocols, and any procedures for monitoring adherence (eg, drug tablet return, laboratory tests)	_______12______
	11d	Relevant concomitant care and interventions that are permitted or prohibited during the trial	_______12______
Outcomes	12	Primary, secondary, and other outcomes, including the specific measurement variable (eg, systolic blood pressure), analysis metric (eg, change from baseline, final value, time to event), method of aggregation (eg, median, proportion), and time point for each outcome. Explanation of the clinical relevance of chosen efficacy and harm outcomes is strongly recommended	_______5/6_____
Participant timeline	13	Time schedule of enrolment, interventions (including any run-ins and washouts), assessments, and visits for participants. A schematic diagram is highly recommended (see Figure)	_____13/14____

For peer review only - http://bmjopen.bmj.com/site/about/guidelines.xhtml

Sample size	14	Estimated number of participants needed to achieve study objectives and how it was determined, including clinical and statistical assumptions supporting any sample size calculations	____16_______
Recruitment	15	Strategies for achieving adequate participant enrolment to reach target sample size	_____9/10_____
**Methods: Assignment of interventions (for controlled trials)**			
Allocation:			
Sequence generation	16a	Method of generating the allocation sequence (eg, computer-generated random numbers), and list of any factors for stratification. To reduce predictability of a random sequence, details of any planned restriction (eg, blocking) should be provided in a separate document that is unavailable to those who enrol participants or assign interventions	_____10______
Allocation concealment mechanism	16b	Mechanism of implementing the allocation sequence (eg, central telephone; sequentially numbered, opaque, sealed envelopes), describing any steps to conceal the sequence until interventions are assigned	_____10______
Implementation	16c	Who will generate the allocation sequence, who will enrol participants, and who will assign participants to interventions	_____9/10____
Blinding (masking)	17a	Who will be blinded after assignment to interventions (eg, trial participants, care providers, outcome assessors, data analysts), and how	______6______
	17b	If blinded, circumstances under which unblinding is permissible, and procedure for revealing a participant's allocated intervention during the trial	______N/A____
**Methods: Data collection, management, and analysis**			
Data collection methods	18a	Plans for assessment and collection of outcome, baseline, and other trial data, including any related processes to promote data quality (eg, duplicate measurements, training of assessors) and a description of study instruments (eg, questionnaires, laboratory tests) along with their reliability and validity, if known. Reference to where data collection forms can be found, if not in the protocol	_______15_____
	18b	Plans to promote participant retention and complete follow-up, including list of any outcome data to be collected for participants who discontinue or deviate from intervention protocols	_______14_____

Data management	19	Plans for data entry, coding, security, and storage, including any related processes to promote data quality (eg, double data entry; range checks for data values). Reference to where details of data management procedures can be found, if not in the protocol	_____15______
Statistical methods	20a	Statistical methods for analysing primary and secondary outcomes. Reference to where other details of the statistical analysis plan can be found, if not in the protocol	_____15______
	20b	Methods for any additional analyses (eg, subgroup and adjusted analyses)	_____15______
	20c	Definition of analysis population relating to protocol non-adherence (eg, as randomised analysis), and any statistical methods to handle missing data (eg, multiple imputation)	_____15______
**Methods: Monitoring**			
Data monitoring	21a	Composition of data monitoring committee (DMC); summary of its role and reporting structure; statement of whether it is independent from the sponsor and competing interests; and reference to where further details about its charter can be found, if not in the protocol. Alternatively, an explanation of why a DMC is not needed	_____17______
	21b	Description of any interim analyses and stopping guidelines, including who will have access to these interim results and make the final decision to terminate the trial	____16_______
Harms	22	Plans for collecting, assessing, reporting, and managing solicited and spontaneously reported adverse events and other unintended effects of trial interventions or trial conduct	____14_______
Auditing	23	Frequency and procedures for auditing trial conduct, if any, and whether the process will be independent from investigators and the sponsor	_____15______
**Ethics and dissemination**			
Research ethics approval	24	Plans for seeking research ethics committee/institutional review board (REC/IRB) approval	_____17______
Protocol amendments	25	Plans for communicating important protocol modifications (eg, changes to eligibility criteria, outcomes, analyses) to relevant parties (eg, investigators, REC/IRBs, trial participants, trial registries, journals, regulators)	_____17______

For peer review only - http://bmjopen.bmj.com/site/about/guidelines.xhtml

Consent or assent	26a	Who will obtain informed consent or assent from potential trial participants or authorised surrogates, and how (see Item 32)	_____9/10____
	26b	Additional consent provisions for collection and use of participant data and biological specimens in ancillary studies, if applicable	____N/A______
Confidentiality	27	How personal information about potential and enrolled participants will be collected, shared, and maintained in order to protect confidentiality before, during, and after the trial	_____15______
Declaration of interests	28	Financial and other competing interests for principal investigators for the overall trial and each study site	_____19______
Access to data	29	Statement of who will have access to the final trial dataset, and disclosure of contractual agreements that limit such access for investigators	_____15______
Ancillary and post-trial care	30	Provisions, if any, for ancillary and post-trial care, and for compensation to those who suffer harm from trial participation	_____14______
Dissemination policy	31a	Plans for investigators and sponsor to communicate trial results to participants, healthcare professionals, the public, and other relevant groups (eg, via publication, reporting in results databases, or other data sharing arrangements), including any publication restrictions	_____17/18____
	31b	Authorship eligibility guidelines and any intended use of professional writers	_____N/A_____
	31c	Plans, if any, for granting public access to the full protocol, participant-level dataset, and statistical code	_____15______
**Appendices**			
Informed consent materials	32	Model consent form and other related documentation given to participants and authorised surrogates	____N/A_______
Biological specimens	33	Plans for collection, laboratory evaluation, and storage of biological specimens for genetic or molecular analysis in the current trial and for future use in ancillary studies, if applicable	____N/A_______

*It is strongly recommended that this checklist be read in conjunction with the SPIRIT 2013 Explanation & Elaboration for important clarification on the items. Amendments to the protocol should be tracked and dated. The SPIRIT checklist is copyrighted by the SPIRIT Group under the Creative Commons "Attribution-NonCommercial-NoDerivs 3.0 Unported" license.

BMJ Open

Graves-PCD: Protocol for a randomised, dose-finding, adaptive trial of the plasma cell depleting agent daratumumab in severe Graves' disease

Journal:	*BMJ Open*
Manuscript ID	bmjopen-2023-079158.R2
Article Type:	Protocol
Date Submitted by the Author:	23-May-2024

Complete List of Authors:	Wolstenhulme, Faye; Newcastle University, Newcastle Clinical Trials Unit, 1-4 Claremont Terrace<br>Bibby, Irena; Newcastle University, Newcastle Clinical Trials Unit, 1-4 Claremont Terrace<br>Cole, Michael; Newcastle University, Population Health Sciences Institute, 4th Floor Ridley Building 1, Queen Victoria Road<br>Grixti, Lydia; Newcastle University, Translational and Clinical Research Institute, Faculty of Medical Sciences, Biomedicine West, International Centre for Life,<br>McGregor, Naomi; Newcastle University, Newcastle Clinical Trials Unit, 1-4 Claremont Terrace<br>Bradley, Penny; Newcastle Upon Tyne Hospitals NHS Foundation Trust, Pharmacy Clinical Trials<br>Maier, Rebecca; Newcastle University, Newcastle Clinical Trials Unit, 1-4 Claremont Terrace<br>Walker, Jenn; Newcastle University, Newcastle Clinical Trials Unit, 1-4 Claremont Terrace<br>Pearce, Simon H; Newcastle University, Translational and Clinical Research Institute, Faculty of Medical Sciences, Biomedicine West, International Centre for Life,<br>Wason, James; Newcastle University, Population Health Sciences Institute, Newcastle University, 4th Floor Ridley Building 1, Queen Victoria Road,

**Primary Subject Heading**:	Diabetes and endocrinology
Secondary Subject Heading:	Research methods
Keywords:	Patients, General endocrinology < DIABETES & ENDOCRINOLOGY, Thyroid disease < DIABETES & ENDOCRINOLOGY, Clinical Trial

SCHOLARONE™
Manuscripts

Graves-PCD: Protocol for a randomised, dose-finding, adaptive trial of the plasma cell depleting agent daratumumab in severe Graves' disease

Authors

Faye Wolstenhulme[1], Irena Bibby[1], Michael Cole[2], Lydia Grixti[3], Naomi McGregor[1], Penny Bradley[4], Rebecca Maier[1], Jenn Walker[1], Simon H Pearce[3], James Wason[2]

[1] Newcastle Clinical Trials Unit, Newcastle University, 1-4 Claremont Terrace, Newcastle upon Tyne, NE2 4AE

[2] Population Health Sciences Institute, Newcastle University, 4th Floor Ridley Building 1, Queen Victoria Road, Newcastle upon Tyne, NE1 7RU, UK

[3] Translational and Clinical Research Institute, Faculty of Medical Sciences, Biomedicine West, International Centre for Life, Newcastle University, Newcastle upon Tyne, NE1 3BZ

[4] Pharmacy Dept. Royal Victoria Infirmary, Queen Victoria Road, Newcastle upon Tyne, NE1 4LP

Correspondence to james.wason@newcastle.ac.uk

ORCiD IDs

FW: 0000-0002-4086-7931 IB: 0009-0007-3105-9864 MC: 0000-0003-3426-3717
NM: 0000-0002-5961-124X RM: 0000-0002-7350-3288 JWal: 0000-0002-7442-3060
SHP: 0000-0001-8384-8063, JWas: 0000-0002-4691-126X

Word count: Abstract 285, Text 3779, Tables 5, Figures 1.

For peer review only

ABSTRACT

Introduction: Severe Graves' disease is a life-changing condition with poor outcomes from currently available treatments. It is caused by directly pathogenic thyroid stimulating hormone (TSH) receptor stimulating antibodies (TRAb), which are secreted from plasma cells. The human anti-CD38 monoclonal antibody daratumumab was developed to target plasma cells which express high levels of CD38, and is currently licensed for treatment of the plasma cell malignancy, myeloma. However, it can also deplete benign plasma cells with the potential to reduce TRAb and alter the natural history of severe Graves' disease. This study aims to establish proof of concept that daratumumab has efficacy in severe Graves' disease patients and will provide important data to inform a choice of dosing regimen for subsequent trials.

Methods and Analysis: The Graves-PCD trial aims to determine if daratumumab modulates the humoral immune response in patients with severe Graves' disease, and if so, over what time period, and to find an optimal dose. It is a single blinded, randomised, dose-finding, adaptive trial using 4 different doses of daratumumab or placebo in 30 adult patients. Part 1 of the trial is dose-finding and, following an interim analysis; in part 2 the remaining patients will be randomised between the chosen dose(s) from the interim analysis or placebo. The primary outcome is the percentage change in serum TRAb antibodies from baseline to 12 weeks.

Ethics and Dissemination

The trial received a favourable ethical opinion from London - Hampstead Research Ethics Committee (reference 21/LO/0449). The results of this trial will be disseminated at international meetings, in the peer-reviewed literature and through partner patient group newsletters and presentations at patient education events.

Registration

ISRCTN81162400

For peer review only

STRENGTHS AND LIMITATIONS OF THIS STUDY

- Current treatments for patients with severe Graves' disease are unsatisfactory and there is therefore significant unmet clinical need for new treatment
- Adaptive design allows efficient investigation of multiple doses
- The long-term remission of hyperthyroidism cannot be assessed in the 24 week follow-up period
- The duration of treatment or dosing intervals will not be tested in this study

INTRODUCTION

Graves' disease (autoimmune hyperthyroidism) affects around 3% of women and 0·5% of men, and most commonly presents in people in their 30s and 40s with a disproportionate burden of ill-health falling on working-age women.(1 ,2) Typical symptoms include weight loss, palpitations, heat intolerance, tremor, breathlessness, sweating, insomnia, loss of concentration and irritability. Around 40% of patients develop Thyroid Eye Disease (TED),(3) which can cause facial disfigurement as well as functional visual problems and even loss of sight. Quality of life in TED patients is poor, worse than for diabetes, and similar to patients with inflammatory bowel disease.(4) Rarely, Thyroid Dermopathy (Pretibial Myxoedema) may also occur leading to a brawny thickening of the skin on the lower legs and feet.(5) These latter 2 problems produce particularly distressing symptoms for a relatively young and active patient group. Around 10% of Graves' patients have a severe form of the disease, and experience severe thyrotoxicosis (serum FT4 ≥50pmol/l [upper limit of reference range 22pmol/l] or FT3≥15pmol [upper limit of reference range 6.8pmol/L]), failure of medical control of hyperthyroidism, large goitre, TED of sufficient severity to affect visual function or Thyroid Dermopathy. Current treatments for these patients with severe Graves' disease give unsatisfactory outcomes and are expensive, typically involving surgical thyroidectomy with or without several episodes of eye surgery.(6)

For peer review only

Conventional treatment of Graves' disease with antithyroid drugs (carbimazole, methimazole or propylthiouracil) leads to remission for only around 50% of patients, decreasing to less than 20% for patients with severe disease, and does not improve eye symptoms.(7 ,8) Other treatment options of radio-active iodine or surgical thyroidectomy result in life-long hypothyroidism requiring levothyroxine therapy, which patients are often unwilling to accept.(9) Better treatment options are sorely needed for severe Graves' disease.

Severe Graves' disease is caused by high titres of directly pathogenic thyroid stimulating hormone receptor stimulating antibodies (TRAb), which are secreted from terminally differentiated B lymphocytes known as plasma cells.(10) Not only do TRAbs directly stimulate thyroid hormone over-production (leading to hyperthyroidism), they also cause thyrocyte hyperplasia leading to goitre, and bind to TSH receptors on the surface of orbital fibroblasts leading to mitogenic signalling that triggers and perpetuates eye disease. Failure of medical control and/or early relapse following conventional antithyroid drugs reflects the persistence of these long-lived, TRAb-secreting plasma cells in the secondary lymphoid tissues and bone marrow.

Both benign and malignant plasma cells express high levels of the cell-surface glycoprotein CD38. Daratumumab, a biological drug that targets CD38, was recently licenced for the plasma cell malignancy, myeloma.(11 ,12) This human anti-CD38 monoclonal antibody (mAb) has the potential to deplete plasma cells and therefore produce a rapid reduction in TRAb levels, which may alter the natural history of severe Graves' disease. This study aims to establish proof of concept that daratumumab has efficacy in severe Graves' disease patients and will provide important data to inform a choice of dosing regimen.

Tolbert et al. first reported the use of daratumumab in the treatment of benign, antibody-mediated haematological autoimmune disorders in 2016.(13) In this case a female in her late teens with autoimmune haemolytic anaemia (post stem cell transplant) was successfully treated with daratumumab and had an instant improvement in haemolysis. There are now numerous additional reports of daratumumab being repurposed to target benign disorders.(14-17)

For peer review only - http://bmjopen.bmj.com/site/about/guidelines.xhtml

Daratumumab is licenced for use in myeloma at a dose of 16mg/kg, however, patients with Graves' disease have several orders of magnitude fewer plasma cells and it is therefore expected that lower doses of drug will be active in this patient group with benign disease. To address this question the first stage of the study will be dose-finding. Notably, dose-finding trials in myeloma patients showed no difference in adverse event rates between doses of 8mg/kg and 16mg/kg, and no dose-limiting toxicity up to 24mg/kg.(11) Therefore, in order to define a signal for efficacy, this study will use 9mg/kg as the top dose in stage 1, with reducing concentrations (3mg/kg, 1mg/kg and 0.5mg/kg) along with placebo to determine the dose response in stage 1. As myeloma trials did not find increased adverse events or toxicities at higher doses, these four dose allocations will take place in parallel. Stage 2 of the study will randomise additional patients to one or more optimal doses of daratumumab, as selected from stage 1, or placebo to gain further information about real-life effect size, safety and tolerability.

METHODS AND ANALYSIS

This study protocol is based on the Standard Protocol Items: Recommendations for Interventional Trials (SPIRIT) 2013 statement.(18)

Sponsor and Trial Management

The trial Sponsor is the Newcastle upon Tyne Hospitals NHS Foundation Trust (Email: tnu-tr.sponsormanagement@nhs.net). Trial management is delegated to Newcastle Clinical Trials Unit, Newcastle University.

Objectives

The primary objective is to determine if daratumumab modulates the humoral immune response in patients with severe Graves' disease, with the primary outcome measure the percentage change in serum TRAb antibodies from baseline to 12 weeks. Secondary objectives include determining the optimal dose of daratumumab, assessing safety, patient quality of life and impact on eye symptoms, thyroid function and goitre size (see Table 1).

For peer review only

For peer review only - http://bmjopen.bmj.com/site/about/guidelines.xhtml

Table 1: Objectives and Outcome Measures

	Objectives	Outcome Measures
**Primary**	To determine if daratumumab modulates the humoral immune response in Graves' disease patients	Change in serum TRAb antibodies from baseline to 12 weeks compared to change in placebo group
**Secondary**	To determine how fast daratumumab modulates the humoral immune response in Graves' disease patients	Change in serum TRAb antibodies from baseline to 2, 4, 6, 12 and 24 weeks
	To determine the optimal dose (or dose range) of daratumumab for Graves' disease patients	Dose-response curve for daratumumab against change in serum TRAb antibodies from baseline to 6 and 12 weeks
	To determine if daratumumab reduces thyroid hormone levels	Change in serum FT3 and FT4 from baseline to 2, 4, 6, 12 and 24 weeks
	To determine if daratumumab changes the time course of serum TSH	Change in serum TSH from baseline to 2, 4, 6, 12 and 24 weeks
	To determine if daratumumab changes thyroid size	Change in thyroid volume from baseline to 24 weeks measured by ultrasound
	To determine if daratumumab changes other thyroid autoantibodies	Change in serum ATPO and thyroglobulin antibodies from baseline to 6, 12 and 24 weeks
	To determine if daratumumab improves thyroid eye disease	Change in CAS, composite eye index and GOQoL score from baseline to 6, 12 and 24 weeks
	To determine if daratumumab improves thyroid symptom-related QoL	Change in ThyPRO39 score from baseline to 6, 12 and 24 weeks
	To determine if daratumumab is safe in this patient group	Change in serum immunoglobulins, specific antibodies including (SARS-CoV2) and blood count parameters from baseline to 6, 12 and 24 weeks.<br>Adverse Reactions to 24 weeks
**Experimental**	To determine if daratumumab changes lymphocyte/plasma cell transcriptomic markers	Analysis of blood plasma cell markers and mRNA signature
	To determine if daratumumab changes the lymphocyte subsets	Change in lymphocyte subsets (by FACS) from baseline to 6, 12 and 24 weeks

Footnotes: TRAb- TSH receptor antibodies; CAS- clinical activity score; GOQoL- Graves' orbitopathy quality of life instrument; ThyPRO39- thyroid patient reported outcome; FACS- fluorescent-activated cell sorting.

Trial Design

For peer review only

For peer review only - http://bmjopen.bmj.com/site/about/guidelines.xhtml

This is an adaptive, 2-stage randomised phase IIa, single centre clinical trial that will aim to recruit 30 patients with severe Graves' disease from secondary care at the Newcastle upon Tyne Hospitals NHS Foundation Trust. It is a single blinded trial in which participants will be blind to allocation. Recruitment start date: October 2021; planned end of trial (last patient last visit) April 2024.

Stage 1 is a dose-response study using 4 doses of daratumumab (9mg/kg, 3mg/kg, 1mg/kg, 0.5mg/kg) and a colourless, volume-matched placebo infusion in approximately 15 patients (i.e. five groups of n=3, randomised in a 1:1:1:1:1 ratio using permuted random blocks without stratification) Figure 1.

Following Stage 1, an interim analysis will be performed of the first 15 patients in order to select an optimal dose(s) of daratumumab for Stage 2. The dose selection will be based on an analysis of the reduction in TRAb antibody concentration and safety assessed at 12 weeks. In stage 2, the remaining 15 patients will be randomised between placebo and one or two chosen doses of daratumumab depending on results of the interim analysis. Patients in both stages of the study will have all the same assessments and will be followed up for 24 weeks, with the primary endpoint being measured at week 12.

Figure 1: Design of study

Inclusion Criteria

1. Patients ≥18yrs old
2. Recent-onset Graves' disease (within 12 months) (defined as date of first thyroid function test showing hyperthyroidism (FT4 and TSH) in current episode).
3. TRAb antibody concentrations above 10U/L (on Roche or Brahms TRAb binding assays)
4. One or more of:
    - Pre-treatment severe hyperthyroidism (FT4 ≥50 pmol/L; or FT3 ≥15 pmol/l)
    - Persisting hyperthyroidism despite more than 12 weeks of antithyroid drug therapy (defined as FT3 above the upper limit of the reference range following 12 weeks of carbimazole treatment at a dose of 40mg or more daily (or equivalent dose of PTU))

For peer review only

- Inflammatory thyroid eye disease (defined as clinical activity score, CAS≥3), or thyroid dermopathy
- Large (visible) goitre (WHO grade III)
- 2 or more relapses (3 episodes in total) despite completing 12 months or more of medical treatment on each occasion. Relapse is defined as FT3 above the upper limit of the local reference range.

5. For women of child-bearing potential, willing to use a highly effective contraceptive method during their participation in the trial.
6. Able to understand and speak sufficient English to complete trial procedures
7. Willing and able to provide informed consent prior to any trial procedures taking place

Exclusion Criteria

1. Previous thyroidectomy, or radioiodine treatment within 2 years
2. Pregnant or breastfeeding, or with a plan for pregnancy within 6 months
3. Previous shingles, known untreated cervical dysplasia, hepatitis B & C or HIV infection
4. Anaemia (Hb ≤ 100g/L), thrombocytopenia (≤75 x10^{9}/L) or neutropenia (≤1.0 x10^{9}/L)
5. Known chronic obstructive pulmonary disease (COPD) (defined as a forced expiratory volume [FEV] in 1 second <60% of predicted normal), persistent asthma, or a history of asthma within the last 2 years (intermittent asthma without hospitalisation is allowed)
6. Any significant physical or mental health condition that impacts the safety of the intervention, the interpretation of thyroid function or the ability of a participant to attend for intervention and safety monitoring, eg. major cardiorespiratory disease, renal or hepatic failure, pancreatitis, cancer undergoing active treatment (excluding non-melanoma skin cancer), untreated chronic infection including TB, psychosis, depression impairing Activities of Daily Living
7. Current use of immunosuppressive therapy for thyroid eye disease or other conditions (within 3 months)
8. Current or previous participation in a CTIMP research study within 4 months
9. Hypersensitivity or anaphylactic reaction to previous monoclonal antibody treatments or methylprednisolone
10. Inability, in the opinion of the investigator, to be able to complete the clinical trial visits or procedures.

For peer review only

For peer review only - http://bmjopen.bmj.com/site/about/guidelines.xhtml

Risk Assessment

Daratumumab is a licenced product for myeloma patients and the main risk is that it will have a different side effect profile in patients with benign disease. The typical risks of daratumumab when used for myeloma include transient infusion reactions and haematological dyscrasias, the latter being accounted for by the bone marrow suppression in myeloma patients and leading to a small risk of infection (≤5%).

Daratumumab interferes with urgent blood cross matching and all patients will have a Coombs test and blood group and transfusion antibody screen prior to administration of daratumumab, as well as receiving an information card in case blood transfusion is needed. There is a potential for reactivation of latent viral illness, and so all participants will be screened for hepatitis B, C and HIV infection, and participants with previous shingles or current untreated cervical dysplasia will be excluded. There are no specific risks demonstrated in the Graves' disease population who will be younger and in better overall health than the patient group who currently receive daratumumab for myeloma.

Participants will be offered a course of SARS-CoV2 vaccination prior to treatment with IMP. If loss of immunity to specific pathogens is detected at the end of the study, the participants may be offered repeat vaccination for tetanus, pneumococcus and SARS-CoV2, where clinically appropriate. Throughout their participation in the study, patients will continue their regular "background" medication for treatment of thyrotoxicosis (i.e. antithyroid drugs, with or without levothyroxine) as prescribed by their clinician as per usual clinical care and will be carefully monitored with regular blood tests throughout the study.

Patient Identification, Consent and Screening

Potential participants with severe Graves' disease within secondary care will be identified and approached in person, by post or email with a trial participant information sheet (PIS) by one of the clinical team responsible for their usual management. Potential participants will also be identified and referred by local secondary care Patient Identification Centre sites, or can self-refer to the trial via adverts through organisations such as the British Thyroid Foundation (BTF). Consent discussions and written informed consent will take place in person at a research specific clinic appointment, with the principal investigator (PI) or alternative

For peer review only - http://bmjopen.bmj.com/site/about/guidelines.xhtml

member of the research team who is medically qualified and appropriately trained. For women of child-bearing potential, consent discussions will include the need to use a highly effective form of contraception (as listed on the PIS) for the duration of their participation in the trial. Consent discussions will be documented in medical notes.

Following written informed consent, eligibility for the trial will be assessed by:

- Demographics and medical history including medication history
- Full external medical examination, including weight, heart (including resting pulse rate and 12 lead ECG), chest, abdomen, goitre size assessment, skin, eyes and neurological examination
- Blood tests (FBC, U&E, LFT, HBsAg, HCV antibodies, HIV, Bone chemistry, TRAb antibodies, serum FT4, FT3, TSH, serum IgGAM, Coombs, blood group and red cell phenotyping)
- A urine sample pregnancy test for all female participants of child-bearing potential (to be repeated where there is >14 days between screening and baseline visits)

The trial screening log will include reasons for screen fails and any reasons given for declining the trial.

Randomisation

Randomisation will be performed by delegated and trained members of the site research team using the online system "Sealed Envelope"; a central, secure, 24-hour web-based randomisation system. In both stages, the allocation sequence will be computer-generated using a random permuted block design. Patients will be randomly allocated equally to the available daratumumab doses or to a matched placebo; for example, if 2 doses of daratumumab plus placebo are to be used, the allocation would be 1:1:1. There are no stratification factors.

In Stage 1, patients will be allocated to receive a dose of daratumumab (9, 3, 1, 0.5mg/Kg) or matched placebo. In Stage 2, patients will be allocated to receive daratumumab (one or two dose levels as determined by the interim analysis), or matched placebo.

Intervention

Each participant will receive 2 infusions, on day 0 and day 14 (+/-3 days). The prescribed daratumumab dose will be prepared in a 500 or 1000ml bag of sodium chloride 0.9%, using

For peer review only - http://bmjopen.bmj.com/site/about/guidelines.xhtml

the most recent available participant weight to calculate banded dose (see Table 2 for volume and infusion rate). Each participant will be prescribed the same banded dose for both infusions, using the same weight for both. Where placebo is allocated, a volume-matched bag of sodium chloride 0.9% will be used. All participants will be pre-medicated with three non-investigational medicinal products (NIMPs), paracetamol (1g po), methylprednisolone (100mg IV) and chlorphenamine (10mg IV) at least 30 minutes prior to their allocated infusion.

Table 2: Infusion Rate Table

Time (Hr:Min)	Rate (mls /Hr)	Total volume at end of time (ml)
**Stage 1 (1000mls)**		
1	80	80
2	160	240
3	240	480
4	320	800
4:40	320	1000
**Stage 2 rate A (1000mls)**		
1	50	50
2	100	150
3	150	300
4	200	500
5	200	700
6	200	900
6:30	200	1000
**Stage 2 rate B (500mls)**		
1	50	50
2	100	150
3	150	300
4	200	500

Table 2: The maximum infusion rate of daratumumab was determined by the recommendation of the SmPC for daratumamab (DARZALEX 20 mg/mL concentrate for solution for infusion). For Stage 1, all participants will receive the IMP/placebo diluted in 1000mls sodium chloride 0.9% for both infusions. For stage 2, some patients may receive higher doses of daratumumab, so a more prolonged first infusion will be given to all participants. If participants have no IRRs during their first dose in stage 2, then the second dose may be given in 500mls sodium chloride 0.9% with a faster infusion rate, in line with SmPC recommendations.

Vital signs (pulse rate, blood pressure and temperature) will be recorded prior to the start of each infusion and every 15 minutes during the course of the infusion, and for 1 hour afterwards. Infusion related reactions will be addressed as shown in Table 3, and documented as an adverse event. Infusion charts will be completed to document compliance with

For peer review only - http://bmjopen.bmj.com/site/about/guidelines.xhtml

prescribed dose. Participants will be followed up by phone approximately 2 days after each infusion visit to check for any adverse events. After the day 14 visit, no further IMP will be administered. Patients will continue their regular medications, including antithyroid drugs and betablockers throughout the study, as deemed appropriate by the clinical team.

Table 3: Infusion Related Reactions

**Grade 4 reactions**	Life-threatening	Stop and permanently discontinue infusion.
**Grade 3 reactions**	Severe, including:<br>• Tachycardia ≥120BPM or drop in SBP ≥30mmHg over pre-infusion with symptoms<br>• Rise in SBP ≥30mmHg over pre-infusion or tachypnoea ≥24/min<br>• Hypoxia SaO2 <92%	Stop infusion until observations improve and restart at 50% of the infusion rate at which IRR occurred. If the patient does not experience additional symptoms, infusion rate escalation may be resumed at increments and intervals as appropriate. The procedure above should be repeated in the event of recurrence of Grade 3 symptoms. After the infusion re-start attempts, the IMP treatment will be permanently discontinued upon the third occurrence of a Grade 3 or greater IRR.
**Grade 1-2 reactions**	Mild to moderate, including:<br>• nasal congestion<br>• cough<br>• chills<br>• throat irritation<br>• vomiting<br>• nausea	Stop infusion in case of clinically significant reaction only until observations improve and restart at 50% of the infusion rate at which IRR occurred. If the patient does not experience any further IRR symptoms, infusion rate escalation may be resumed at increments and intervals as clinically appropriate up to the maximum rate. Infusion does not need to be stopped if IRR is not clinically significant.

Assessments

Follow up visits will take place at weeks 4, 6, 12 and 24 (+/- 1 week); the details of assessments are provided in Table 4. At each visit, concomitant medications will be recorded, an adverse event check will be performed, compliance with anti-thyroid medication checked and full blood count and thyroid function (TSH, FT4, FT3 and TRAb) will be assessed. Other assessments include goitre size assessment, eye exam, patient reported quality of life measures (GOQoL(4) and ThyPRO(19), specific antibody tests and other safety bloods (FBC,

For peer review only - http://bmjopen.bmj.com/site/about/guidelines.xhtml

LFT). At the end of their participation in the trial, participants will be asked their opinion on which treatment allocation they received, and if they would recommend their treatment to a friend with the same condition. In addition, we plan to undertake exploratory analyses of lymphocyte subsets determined by flow cytometry and transcriptomic markers of plasma cells using qRT-PCR.(20)

Table 4: Schedule of Assessments

**Assessment/Activity**	**Visit 1**	**Visits 2 & 3**	**Visit 4**	**Visit 5**	**Visit 6**	**Visit 7**	**Visit 8**	**Visit 9**
			**Day 0**	**Day 14 (+/-3 days)**	**4 weeks (+/- 1 week)**	**6 weeks (+/- 1 week)**	**12 weeks (+/- 1 week)**	**24 weeks (+/- 1 week)**
Written Informed Consent	X							
Demographics & medical history	X							
Physical exam	X							
12-lead ECG	X							
Eye exam (CAS)	X							
Hep B & C serology, HIV	X							
Bone chem	X							
Coombs, blood group, RBC phenotyping	X							
Pregnancy testing (females)	X						X	
COVID vaccination offered		X X						
IMP infusion			X	X				
Weight	X	X	X	X	X	X	X	X
Resting pulse rate	X		X	X	X	X	X	X
Goitre size assessment	X		X			X	X	X
FBC	X		X	X	X	X	X	X
TSH, FT4, FT3, TRAb	X		X	X	X	X	X	X
U&E, LFT	X		X	X	X	X		
IgGAM	X		X			X	X	X
Height			X					
Eye exam (composite eye index)			X			X	X	X
Thyroid volume USS			X					X
QoL questionnaires (GOQoL, ThyPRO)			X			X	X	X
TgAb, ATPO			X			X	X	X
Specific antibodies			X				X	X
Exploratory analysis sample			X			X	X	X
Vital signs			X	X				
Compliance with ATD			X	X	X	X	X	X
Concomitant medication		X	X	X	X	X	X	X
Adverse event reporting		X	X	X	X	X	X	X
Follow-up phone call			X	X				
End of trial questions								X

For peer review only - http://bmjopen.bmj.com/site/about/guidelines.xhtml

Discontinuation of Treatment and Withdrawal

Participants who have completed one or more daratumumab/placebo infusions may remain on the trial but choose to discontinue treatment. These participants will continue to take part in the trial assessments as per the trial schedule of events. Alternatively, an investigator may discontinue a participant from receiving trial treatment at any time (until administration of second infusion is completed), if the investigator considers it necessary for any reason. Participants who withdraw from the trial after receiving IMP (full or part dose) will not be replaced. Participants who withdraw before IMP is given will be replaced.

Safety Reporting

Adverse events (AEs) will be documented in patient medical notes -before entry into the trial database. AEs judged by the investigator responsible for patient care as consistent with the usual clinical pattern for patients with severe Graves' disease (e.g. Hyperthyroidism, hypothyroidism, tachycardia, bradycardia) will not be reported. Disease progression will be recorded as an AE only if it is more severe than expected in the trial population. AEs will be coded according to MedDRA classifications. All AEs meeting the criteria for seriousness (SAEs) will be reported by the investigator to the trial Sponsor within 24 hours of awareness, and suspected unexpected serious adverse reactions will be reported to the MHRA and research ethics committee (REC) by the trial Sponsor in line with regulatory reporting timelines.

End of Trial

The end of the trial is defined as last patient last visit (LPLV). Treatment allocation will be revealed to all participants (and their GP/referring clinician where applicable) by letter at the end of the trial, with contact details to discuss if required. At the end of their participation in the study participants will return to standard NHS care.

Payment

No payment will be made to participants for taking part in this trial, although reasonable travel expense reimbursement including taxis will be made for trial visits.

Data Management

For peer review only

For peer review only - http://bmjopen.bmj.com/site/about/guidelines.xhtml

The Clinical Data Management System (CDMS) used to capture trial data is Red Pill, supplied by Sealed Envelope. Users have individual password protected user accounts appropriate to their trial duties (e.g. randomisation, data entry, monitor). Patients are identified on the CDMS using a unique participant ID. Data monitoring will include a mixture of central, off-site and on-site approaches and will be outlined in a risk-based monitoring plan prior to the start of the trial. The trial specific Data Management Plan will include details of how the validity and quality of data will be monitored and managed. As far as possible, validations will be built into the CDMS and additional manual validations will be detailed in the Data Validation Plan. Trial monitoring will be performed by NCTU as per the monitoring plan and the trial is also liable to audit by the trial Sponsor and regulator.

Until publication of the trial results, access to the full dataset will be restricted to the Trial Management Group (TMG) and to authors of the publication. Requests for anonymised data should be directed to the lead author.

Analysis

The primary analysis will assess whether there is a significant dose-response using percentage reduction in serum TRAb concentration from baseline to 12 weeks using the intention-to-treat (ITT) population (participants analysed according to randomisation allocation).

To do this we will calculate a contrast-based test statistic at a one-sided 5% type I error rate.(21) If this test statistic is significant, we will test whether there is a mean difference in the outcome between individual doses and placebo using a suitable linear model. We will also perform a simple test of all placebo participants vs all daratumumab patients using a linear model that adjusts for presence of eye disease. We will also fit quadratic and three-parameter Emax models to assess dose-response.(22) This analysis will inform selection of the most suitable dose for subsequent trials. Exploratory analyses using subgroups of patients including by gender, smoking status, presence of TED and change in serum TRAb will be undertaken.

Interim Analyses and Criteria for the Premature Termination of the Trial

An interim analysis of dose-response will be conducted after 15 patients have provided 12-week follow-up data. To assess efficacy, a three-parameter Emax model will be fitted to the logarithm of the percentage change in TRAb from baseline to week 12 using the DoseFinding package in R(23 ,24) in the dose-response population (participants analysed according to the

For peer review only - http://bmjopen.bmj.com/site/about/guidelines.xhtml

amount of daratumumab received). This model is used to estimate the plateau effect and i) the dose that gives a mean reduction of 50% and ii) the dose that gives 90% of the plateau effect. If the estimated plateau effect corresponds to >50% reduction in TRAb, the trial will continue with placebo and doses (i) and (ii) defined above. If the estimated plateau corresponds to less than 25% reduction in TRAb, the trial will terminate early for lack of promising dose-response unless the DMC agree that there has been an unequivocal improvement in serum thyroid hormone measurements in the active IMP vs placebo groups; if it is between 25%-50% reduction, the trial will continue with doses of 9mg/kg and a higher daratumumab dose (e.g. 12 or 16mg/Kg as determined by safety data), plus placebo. Safety information will be taken into consideration in deciding stage 2 dose(s) and could over-ride the model-based dose recommendations.

Statistical Size Calculations

A 50% or more reduction in TRAb antibody concentration from baseline within 12 weeks of intervention was chosen as biologically plausible and clinically important. An observational study of milder Graves' disease patients showed a mean change in TRAb concentration of 2% over 6 weeks, with an SD of 15%. Assuming: a standard deviation of 25%, to account for the outcome being measured at 12 weeks; a 5% reduction in placebo treated subjects; an Emax dose-response; a plateau 60% reduction in TRAb concentration over 12 weeks with active IMP; and 1mg/kg dose giving half of the total, 27 participants will give over 90% power (5% one-sided type I error) to conclude there is a dose response relationship. In order to allow for 10% drop out, we will plan to recruit 30 participants.

Power was verified through simulations in 'R',(24) assuming the logarithm of the percentage reduction to be normally distributed with SD of 0.5 (equivalent to 25% SD when the mean is 50% reduction). The power/type I error rate for a range of scenarios is given in Table 5.

Table 5: Power/type 1 error rate calculations

Scenario	Probability to conclude dose-response (10000 replicates)
1 – plateau of 80% reduction ED50 =3mg/kg	>99%
2 – plateau of 60% reduction, ED50=1mg/kg	92%
3 – plateau of 60% reduction, ED50=3mg/kg	83%

For peer review only - http://bmjopen.bmj.com/site/about/guidelines.xhtml

4 – null scenario	3.2%

Footnote: The R code required to reproduce these results can be found in supplementary material 1.

Trial Oversight

The TMG will meet approximately monthly throughout the trial, and includes the CI, statisticians, Newcastle Clinical Trials Unit staff, pharmacy and Sponsor representatives. Oversight is provided by an independent Data Monitoring Committee who will meet at the start of the trial, at the interim analysis point and on an ad hoc basis throughout, as well as a Trial Steering Committee (TSC) who will generally meet following a DMC meeting. The terms of reference and composition of these committees are outlined in their respective charters.

Protocol Amendments

NCTU will submit amendments to the HRA and MHRA/REC (as appropriate) as well as informing ISRCTN, trial recruiters, investigators and the relevant R&D department of changes. A list of version changes will be kept in the trial protocol appendix and can be found in supplementary material 2.

Trial Status

This manuscript is based on protocol version 6.0 dated 18 May 2023. The Graves-PCD trial opened to recruitment on 29 September 2021. At the time of writing, stage 2 of the trial is underway.

Patient and Public Involvement

Patients were involved in the design of the trial via the British Thyroid Foundation (www.btf-thyroid.org), and lay members will have oversight and input during the trial and dissemination as members of the TSC.

ETHICS AND DISSEMINATION

The trial received a favourable ethical opinion from London - Hampstead Research Ethics Committee (reference 21/LO/0449). The results of this trial will be disseminated at international meetings, in the peer-reviewed literature and through partner patient group newsletters and presentations at patient education events.

For peer review only

For peer review only - http://bmjopen.bmj.com/site/about/guidelines.xhtml

ACKNOWLEDGEMENTS

We would like to acknowledge the ongoing support provided by the British Thyroid Foundation. We are grateful to Prof. Ruth Andrew of Edinburgh University for advice concerning dose choices and to Dr. Catherine Stroud of the Newcastle upon Tyne Hospitals NHS Foundation Trust for immunological safety advice.

AUTHORS' CONTRIBUTIONS

SHP had the original idea for the trial. The trial protocol was developed by SHP, with substantial input and revision by FW, JWas, MC, RM, JWal, LG and PB. IB and NM also made changes to the protocol at various stages of development. The trial protocol manuscript was primarily drafted by FW, with substantial input from SHP and JWas. All other coauthors approved the final version of the manuscript.

COMPETING INTERESTS STATEMENT

SHP has received speaker fees from Merck, IBSA, and consulted for Apitope/Worg and Immunovant/Roivant. JWas has consulted for Worg. No other author has conflicts of interest to declare.

FUNDING STATEMENT

This trial is funded by the UKRI Medical Research Council (MRC), ref MR/V005898/1

REFERENCES

1. Nyström HF, Jansson S, Berg G. Incidence rate and clinical features of hyperthyroidism in a long-term iodine sufficient area of Sweden (Gothenburg) 2003-2005. *Clin Endocrinol (Oxf)* 2013;**78**(5):768-76.
2. Vanderpump MP. The epidemiology of thyroid disease. *Br Med Bull* 2011;**99**:39-51.
3. Chin YH, Ng CH, Lee MH, et al. Prevalence of thyroid eye disease in Graves' disease: A meta-analysis and systematic review. *Clin Endocrinol (Oxf)* 2020;**93**(4):363-74.
4. Gerding MN, Terwee CB, Dekker FW, et al. Quality of life in patients with Graves' ophthalmopathy is markedly decreased: measurement by the medical outcomes study instrument. *Thyroid* 1997;**7**(6):885-9.
5. Fatourechi V, Pajouhi M, Fransway AF. Dermopathy of Graves disease (pretibial myxedema). Review of 150 cases. *Medicine (Baltimore)* 1994;**73**(1):1-7.
6. Estcourt S, Hickey J, Perros P, et al. The patient experience of services for thyroid eye disease in the United Kingdom: results of a nationwide survey. *Eur J Endocrinol* 2009;**161**(3):483-7.

For peer review only - http://bmjopen.bmj.com/site/about/guidelines.xhtml

7. Struja T, Fehlberg H, Kutz A, et al. Can we predict relapse in Graves' disease? Results from a systematic review and meta-analysis. *Eur J Endocrinol* 2017;**176**(1):87-97.

8. Eckstein AK, Plicht M, Lax H, et al. Thyrotropin receptor autoantibodies are independent risk factors for Graves' ophthalmopathy and help to predict severity and outcome of the disease. *J Clin Endocrinol Metab* 2006;**91**(9):3464-70.

9. van Kinschot CM, Soekhai VR, de Bekker-Grob EW, et al. Preferences of patients and clinicians for treatment of Graves' disease: a discrete choice experiment. *Eur J Endocrinol* 2021;**184**(6):803-12.

10. Smith TJ, Hegedüs L. Graves' Disease. *N Engl J Med* 2016;**375**(16):1552-65.

11. Lokhorst HM, Plesner T, Laubach JP, et al. Targeting CD38 with Daratumumab Monotherapy in Multiple Myeloma. *N Engl J Med* 2015;**373**(13):1207-19.

12. Lonial S, Weiss BM, Usmani SZ, et al. Daratumumab monotherapy in patients with treatment-refractory multiple myeloma (SIRIUS): an open-label, randomised, phase 2 trial. *Lancet* 2016;**387**(10027):1551-60.

13. Tolbert VP, Goldsby R, Huang J, et al. Daratumumab Is Effective in the Treatment of Refractory Post-Transplant Autoimmune Hemolytic Anemia: A Pediatric Case Report. *Blood* 2016;**128**(22):4819-19.

14. Ostendorf L, Burns M, Durek P, et al. Targeting CD38 with Daratumumab in Refractory Systemic Lupus Erythematosus. *N Engl J Med* 2020;**383**(12):1149-55.

15. Migdady Y, Ediriwickrema A, Jackson RP, et al. Successful treatment of thrombocytopenia with daratumumab after allogeneic transplant: a case report and literature review. *Blood Advances* 2020;**4**(5):815.

16. Zand L, Rajkumar SV, Leung N, et al. Safety and efficacy of daratumumab in patients with proliferative GN with monoclonal immunoglobulin deposits. *J Am Soc Nephrol* 2021;**32**(5):1163-73.

17. Scheibe F, Ostendorf L, Reincke SM, et al. Daratumumab treatment for therapy-refractory anti-CASPR2 encephalitis. *J Neurol* 2020;**267**:317-23.

18. Chan A-W, Tetzlaff JM, Gøtzsche PC, et al. SPIRIT 2013 explanation and elaboration: guidance for protocols of clinical trials. *BMJ* 2013;**346**.

19. Watt T, Bjorner JB, Groenvold M, et al. Development of a Short Version of the Thyroid-Related Patient-Reported Outcome ThyPRO. *Thyroid* 2015;**25**(10):1069-79.

20. Streicher K, Morehouse CA, Groves CJ, et al. The plasma cell signature in autoimmune disease. *Arthritis Rheumatol* 2014;**66**(1):173-84.

21. Thomas N. Understanding MCP-MOD dose finding as a method based on linear regression. *Stat Med* 2017;**36**(27):4401-13.

22. Macdougall J. Analysis of Dose–Response Studies—Emax Model. In: Ting N, ed. Dose Finding in Drug Development. New York, NY: Springer New York, 2006:127-45.

23. Pinheiro J, Bornkamp B, Glimm E, et al. Model-based dose finding under model uncertainty using general parametric models. *Stat Med* 2014;**33**(10):1646-61.

24. R Core Team. R: A Language and Environment for Statistical Computing. Vienna, Austria: R Foundation for Statistical Computing, 2024:https://www.R-project.org/.

For peer review only

For peer review only - http://bmjopen.bmj.com/site/about/guidelines.xhtml

Figure 1: Design of study

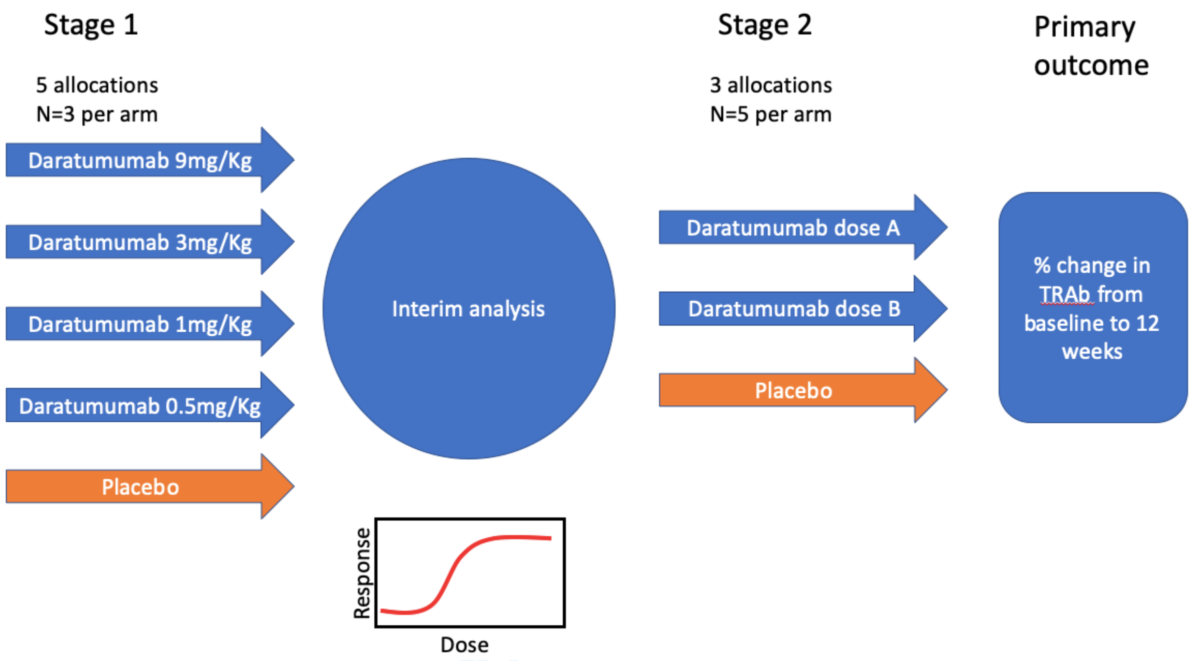

```
library(boot)
library(MCPMod)
library(DoseFinding)


#Assume that -log(percentage of baseline) (-log of ratio of follow-up TRAb to baseline TRAb) is normally distributed and the outcome


#Function to run a given number of simulation replicates:

simulation=function(
  initialdoses=c(0,0.5,1,3,9), #Initial range of doses given
  stage1.nperdose=3, #Initial number of patients per dose in stage 1
  maximumpercentagereduction=80, #plateau percentage reduction from baseline
  minimumpercentagereduction=5,#mean reduction with placebo
  ed50=2,#dose that gives 50% of effect
  errSD=sqrt(1/4),#SD of transformed outcome (assuming the SD is 25 at the mean of 50% reduction) is approximately sqrt(1/50^2 * 25^2)
  alpha=0.05, # the alpha level
nreplicates=1000 #number of simulation replicates to run
)
{
  #define vectors to track statistical properties

  power.contrast=rep(0,nreplicates)
estimated.dose50=rep(NA,nreplicates)
estimated.ed50=rep(NA,nreplicates)

selectedstage2doses=matrix(NA,nreplicates,3)
xdose<-initialdoses
nsam<-stage1.nperdose #number of patient per dose

#Hypothesised dose response

#transform of maximum %age reduction
ehi<- -log((100-maximumpercentagereduction)/100)
#Transform for placebo reduction
elo<- -log((100-minimumpercentagereduction)/100)


for(replicate in 1:nreplicates)
{

#sim first stage data
dfData.stage1 <- genDFdata("emax", c(e0 = elo, eMax = ehi-elo, ed50 = ed50), doses=xdose, sigma=errSD, n=nsam)


#plot(dfData.stage1$dose,dfData.stage1$resp)
#points(aggregate(dfData.stage1$resp, list(dose=dfData.stage1$dose), mean),pch=15)

#fit an ANOVA model to estimate means
fit.aovmean = lm(resp~as.factor(dose)-1, data=dfData.stage1) #no intercept
#summary(fit.aovmean)
EstMean.aov = coef(fit.aovmean) # Extract the estimated sample means
aov.sigma = summary(fit.aovmean)$sigma # Extract the estimated sigma

# PoC: contrast mean >0 statistically?
len.Dosage<-length(xdose)

n=nsam # sample size for each dosage
# The c's for 4-dose and 1-placebo
Contrast.Coef = c(-2,-1,0,1,2)
# Implementation of equation 9.2. with 1-sided test "Dose Finding in Drug Development" Naitee Ting. Springer
# where "qt" is for the quantile-t with one-sided (i.e. 1-alpha)

teststatistic.contrast=sum(Contrast.Coef*EstMean.aov)/(aov.sigma*sqrt(sum(Contrast.Coef^2/n)))
pvalue=1-pt(teststatistic.contrast,df=(n-1)*len.Dosage)
PoC.Contrast=sum(Contrast.Coef*EstMean.aov)/(aov.sigma*sqrt(sum(Contrast.Coef^2/n))) >qt(1-alpha,df=(n-1)*len.Dosage)

# fit the "Emax" model
fitemax = fitMod(dose, resp, data=dfData.stage1, model="emax",  bnds = c(0.01, 25))
# Call "plot" to plot the Emax fit
#plot(fitemax,CI=TRUE,plotData="meansCI",xlab="Dose",ylab="Response")


#function to find dose achieving reponse of 50% and bootstrap CI



#If e0+eMax is less than the equivalent of a 25% reduction, then stop early:

if((fitemax$coefs[1]+fitemax$coefs[2])< (-log(0.75)))
{
  next;
}

#If e0+eMax is between 25% and 50% reduction, then second stage doses should be 0, 3 and 9:

if((fitemax$coefs[1]+fitemax$coefs[2])< (-log(0.5)))
{

  xdose.stage2.overrun<-c(0,0.5,1,3,9)
  dfData.stage2.overrun <- genDFdata("emax", c(e0 = elo, eMax = ehi, ed50 = ed50), doses=xdose.stage2.overrun, sigma=errSD, n=1)
  xdose.stage2.afterinterim=c(0,3,9)

  selectedstage2doses[replicate,]=c(0,3,9)
```

```
}

#If e0+eMax is higher than 50% reduction, then select dose that gives 50% reduction and one that gives 90% of plateau reduction
if((fitemax$coefs[1]+fitemax$coefs[2])>= (-log(0.5)))
{

#extra dose chosen to be close to plateau effect:
plateau=fitemax$coefs[1]+fitemax$coefs[2]

#find dose that gives 90% of plateau effect:

dose.nearplateau=doseforgivenmean(fitemax,mu=0.9*plateau)

dose.nearplateau=round(dose.nearplateau)



#Estimated dose that gives 50% reduction, or 50% of plateau if plateau is lower than 50:

dose.50=doseforgivenmean(fitemax,mu=-log(0.5))
#round to nearest dose:

dose.50=round(dose.50)
#If rounded dose.50 is less than or equal to 0 (can be less if e0 is estimated to be high), set to 0.5:

if(dose.50<=0)
{
  dose.50=0.5
}

#If rounded dose.50 is >9, set to 6:

if(dose.50>9)
{
  dose.50=6
}

#If rounded dose.plateau is <= dose.50, set to dose.50+1:

if(dose.nearplateau<=dose.50)
{
  dose.nearplateau=dose.50+1
}

#If rounded dose.nearplateau is >9, set to 9:

if(dose.nearplateau>9)
{
  dose.nearplateau=9
}



#To allow for overrun, secondstage doses include 5 more recruited under the stage 1 allocation, then remaining allocated 3,3,3 to new
#Note this only makes 29

xdose.stage2.overrun<-c(0,0.5,1,3,9)
dfData.stage2.overrun <- genDFdata("emax", c(e0 = elo, eMax = ehi, ed50 = ed50), doses=xdose.stage2.overrun, sigma=errSD, n=1)
xdose.stage2.afterinterim<-c(0,dose.50,dose.nearplateau)

selectedstage2doses[replicate,]=c(0,dose.50,dose.nearplateau)

}

dfData.stage2.afterinterim <- genDFdata("emax", c(e0 = elo, eMax = ehi, ed50 = ed50), doses=xdose.stage2.afterinterim, sigma=errSD, n=

dfData.combined=rbind(dfData.stage1,dfData.stage2.overrun,dfData.stage2.afterinterim)


#Final analysis:

#fit an ANOVA model to estimate means
fit.aovmean = lm(resp~as.factor(dose)-1, data=dfData.combined) #no intercept
summary(fit.aovmean)
EstMean.aov = coef(fit.aovmean) # Extract the estimated sample means
aov.sigma = summary(fit.aovmean)$sigma # Extract the estimated sigma

#Set up contrast: check if even or odd number of unique doses

ndoses=length(unique(dfData.combined$dose))
if(ndoses==5){Contrast.Coef<-c(-2,-1,0,1,2)}
if(ndoses==6){Contrast.Coef<-c(-5,-3,1,1,3,5)} #notice change in contrast from -3,2,-1,1,2,3 so equal spacing throughout
if(ndoses==7){Contrast.Coef<-c(-3,-2,-1,0,1,2,3)}


n=as.double(table(sort(dfData.combined$dose))) # sample size for each dosage

# Implementation of equation 9.2. with 1-sided test "Dose Finding in Drug Development" Naitee Ting. Springer
# where "qt" is for the quantile-t with one-sided (i.e. 1-alpha)


teststatistic.contrast=sum(Contrast.Coef*EstMean.aov)/(aov.sigma*sqrt(sum(Contrast.Coef^2/n)))
```

```
pvalue=1-pt(teststatistic.contrast,df=2*ndoses)

power.contrast[replicate]=ifelse(pvalue<alpha,1,0)


fitemax = fitMod(dose, resp, data=dfData.combined, model="emax",  bnds = c(0.01, 25))

estimated.dose50[replicate]=doseforgivenmean(fitemax,-log(0.5))


estimated.ed50[replicate]=fitemax$coefs[3]


}

return(list(power.contrast=mean(power.contrast),conclude.doseeffect=power.contrast,estimated.dose50=estimated.dose50,estimated.ed50=es
}




#Function to find dose that gives target response assuming three-parameter emax
doseforgivenmean <- function(model, mu) {


  return((model$coefs[3]*(mu-model$coefs[1]))/(model$coefs[2]+model$coefs[1]-mu))
}


#Function for bootstrapping  #currently not modified to work with transformed outcome
dr50norm.boot <- function(data, indices) {
  y <- fit + e[indices]
  fitemaxboot = fitMod(data$dose, y, model="emax",  bnds = c(0.01, 50))
  est.parm = coef(fitemaxboot)
  ED50 = est.parm["ed50"] # extract the estimated ED50
  EMAX = est.parm["eMax"] # extract the estimated EMAX
  E0 = est.parm["e0"] # extract the estimated E0
  phi <- unname((50-E0)/EMAX)
  DoseR <- unname(phi*ED50/(1-phi))
  return(DoseR)
}

 #Scenario 1: large plateau effect, emax model
set.seed(1)
results.scenario1=simulation(nreplicates=10000,ed50 = 3)
print(c("Median ED50: ",median(results.scenario1$estimated.ed50,na.rm=T)))
print(c("Power: ",results.scenario1$power.contrast))

#Scenario 2: medium plateau effect, emax model
set.seed(1)
results.scenario2=simulation(maximumpercentagereduction = 60,ed50=1,nreplicates=10000 )
print(c("Median ED50: ",median(results.scenario2$estimated.ed50,na.rm=T)))
print(c("Power: ",results.scenario2$power.contrast))


#Scenario 3: medium plateau effect, emax model with ED50 equal to 3
set.seed(1)
results.scenario3=simulation(maximumpercentagereduction = 60,ed50=3,nreplicates=10000 )
print(c("Median ED50: ",median(results.scenario3$estimated.ed50,na.rm=T)))
print(c("Power: ",results.scenario3$power.contrast))


#Scenario 4: null:
set.seed(1)
results.scenario4=simulation(minimumpercentagereduction = 5,maximumpercentagereduction = 5,ed50=100,nreplicates=10000 )
print(c("Median ED50: ",median(results.scenario4$estimated.ed50,na.rm=T)))
print(c("Power: ",results.scenario4$power.contrast))
```

Graves-PCD: Protocol for a randomised, dose-finding, adaptive trial of the plasma cell depleting agent daratumumab in severe Graves' disease

Supplementary Material 2

Table S2: Protocol Version History

Protocol version no.	Details of changes made
6.0	4. Trial Design- update to Figure 2<br>5. Trial Settings update<br>6.1. Update to inclusion criteria 2 and 4<br>7.2.1. Patient Identification- Addition of self-referral participant information<br>8.6. Dosage Schedule & Modifications- Clarification on IRRs<br>Appendix 3- Update to Infusion Rate Tables text and Infusion Related Reactions (IRR) concerning Grade 1-2 reactions management.
5.0	8.6 update to Dosage Schedule & Modifications<br>Appendix 3- 16.3.2 update to Infusion Rate Tables text
4.0	Update to Key Trial Contacts<br>Appendix 3- Update to Dose Banding Tables, Infusion Rate Table and Infusion Related Reactions (IRR)
3.0	Update to Key Trial Contacts<br>Typographical corrections<br>7.1.8 Storage of TRAb sample until data lock at visits 4, 7, 8 and 9<br>7.1.11 Addition of end of trial questions<br>7.7 Removal of reconfirmation of eligibility >6 weeks, addition of re-screening >3 months
2.0	2. Addition of rationale for choice of doses in stage 1<br>6.2 Update to exclusion criterion 6<br>7.1.5 Addition of photography for patients with thyroid eye disease<br>7.12 Removal of use of initials<br>8.3 and 9.3.1 Removal of reference to SmPC section 4.5<br>9.4 Addition of yellow card reporting for NIMPs<br>10.1.4 Clarification on criteria for safety assessment at interim analysis<br>16.3.3 Update to response to IRRs
1.0	Original version

For peer review only

For peer review only - http://bmjopen.bmj.com/site/about/guidelines.xhtml

STANDARD PROTOCOL ITEMS: RECOMMENDATIONS FOR INTERVENTIONAL TRIALS

SPIRIT 2013 Checklist: Recommended items to address in a clinical trial protocol and related documents*

For peer review only

Section/item	Item No	Description	Addressed on page number
**Administrative information**			
Title	1	Descriptive title identifying the study design, population, interventions, and, if applicable, trial acronym	__1_________
Trial registration	2a	Trial identifier and registry name. If not yet registered, name of intended registry	__2_________
	2b	All items from the World Health Organization Trial Registration Data Set	throughout
Protocol version	3	Date and version identifier	__17________
Funding	4	Sources and types of financial, material, and other support	__2_________
Roles and responsibilities	5a	Names, affiliations, and roles of protocol contributors	__1, 19______
	5b	Name and contact information for the trial sponsor	___5________
	5c	Role of study sponsor and funders, if any, in study design; collection, management, analysis, and interpretation of data; writing of the report; and the decision to submit the report for publication, including whether they will have ultimate authority over any of these activities	__17________
	5d	Composition, roles, and responsibilities of the coordinating centre, steering committee, endpoint adjudication committee, data management team, and other individuals or groups overseeing the trial, if applicable (see Item 21a for data monitoring committee)	____17_______

**Introduction**			
Background and rationale	6a	Description of research question and justification for undertaking the trial, including summary of relevant studies (published and unpublished) examining benefits and harms for each intervention	____3-5______
	6b	Explanation for choice of comparators	_____4/5_____
Objectives	7	Specific objectives or hypotheses	_____5/6_____
Trial design	8	Description of trial design including type of trial (eg, parallel group, crossover, factorial, single group), allocation ratio, and framework (eg, superiority, equivalence, noninferiority, exploratory)	_____6______
**Methods: Participants, interventions, and outcomes**			
Study setting	9	Description of study settings (eg, community clinic, academic hospital) and list of countries where data will be collected. Reference to where list of study sites can be obtained	_____6_______
Eligibility criteria	10	Inclusion and exclusion criteria for participants. If applicable, eligibility criteria for study centres and individuals who will perform the interventions (eg, surgeons, psychotherapists)	_____7-9_____
Interventions	11a	Interventions for each group with sufficient detail to allow replication, including how and when they will be administered	_____11/12____
	11b	Criteria for discontinuing or modifying allocated interventions for a given trial participant (eg, drug dose change in response to harms, participant request, or improving/worsening disease)	_____12______
	11c	Strategies to improve adherence to intervention protocols, and any procedures for monitoring adherence (eg, drug tablet return, laboratory tests)	_______12______
	11d	Relevant concomitant care and interventions that are permitted or prohibited during the trial	_______12______
Outcomes	12	Primary, secondary, and other outcomes, including the specific measurement variable (eg, systolic blood pressure), analysis metric (eg, change from baseline, final value, time to event), method of aggregation (eg, median, proportion), and time point for each outcome. Explanation of the clinical relevance of chosen efficacy and harm outcomes is strongly recommended	_______5/6_____
Participant timeline	13	Time schedule of enrolment, interventions (including any run-ins and washouts), assessments, and visits for participants. A schematic diagram is highly recommended (see Figure)	_____13/14____

For peer review only - http://bmjopen.bmj.com/site/about/guidelines.xhtml

Sample size	14	Estimated number of participants needed to achieve study objectives and how it was determined, including clinical and statistical assumptions supporting any sample size calculations	____16_______
Recruitment	15	Strategies for achieving adequate participant enrolment to reach target sample size	_____9/10_____

Methods: Assignment of interventions (for controlled trials)

Allocation:

Sequence generation	16a	Method of generating the allocation sequence (eg, computer-generated random numbers), and list of any factors for stratification. To reduce predictability of a random sequence, details of any planned restriction (eg, blocking) should be provided in a separate document that is unavailable to those who enrol participants or assign interventions	_____10______
Allocation concealment mechanism	16b	Mechanism of implementing the allocation sequence (eg, central telephone; sequentially numbered, opaque, sealed envelopes), describing any steps to conceal the sequence until interventions are assigned	_____10______
Implementation	16c	Who will generate the allocation sequence, who will enrol participants, and who will assign participants to interventions	_____9/10____
Blinding (masking)	17a	Who will be blinded after assignment to interventions (eg, trial participants, care providers, outcome assessors, data analysts), and how	______6______
	17b	If blinded, circumstances under which unblinding is permissible, and procedure for revealing a participant's allocated intervention during the trial	______N/A____

Methods: Data collection, management, and analysis

Data collection methods	18a	Plans for assessment and collection of outcome, baseline, and other trial data, including any related processes to promote data quality (eg, duplicate measurements, training of assessors) and a description of study instruments (eg, questionnaires, laboratory tests) along with their reliability and validity, if known. Reference to where data collection forms can be found, if not in the protocol	_______15_____
	18b	Plans to promote participant retention and complete follow-up, including list of any outcome data to be collected for participants who discontinue or deviate from intervention protocols	_______14_____

For peer review only - http://bmjopen.bmj.com/site/about/guidelines.xhtml

Data management	19	Plans for data entry, coding, security, and storage, including any related processes to promote data quality (eg, double data entry; range checks for data values). Reference to where details of data management procedures can be found, if not in the protocol	_____15______
Statistical methods	20a	Statistical methods for analysing primary and secondary outcomes. Reference to where other details of the statistical analysis plan can be found, if not in the protocol	_____15______
	20b	Methods for any additional analyses (eg, subgroup and adjusted analyses)	_____15______
	20c	Definition of analysis population relating to protocol non-adherence (eg, as randomised analysis), and any statistical methods to handle missing data (eg, multiple imputation)	_____15______
**Methods: Monitoring**			
Data monitoring	21a	Composition of data monitoring committee (DMC); summary of its role and reporting structure; statement of whether it is independent from the sponsor and competing interests; and reference to where further details about its charter can be found, if not in the protocol. Alternatively, an explanation of why a DMC is not needed	_____17______
	21b	Description of any interim analyses and stopping guidelines, including who will have access to these interim results and make the final decision to terminate the trial	____16_______
Harms	22	Plans for collecting, assessing, reporting, and managing solicited and spontaneously reported adverse events and other unintended effects of trial interventions or trial conduct	____14_______
Auditing	23	Frequency and procedures for auditing trial conduct, if any, and whether the process will be independent from investigators and the sponsor	_____15______
**Ethics and dissemination**			
Research ethics approval	24	Plans for seeking research ethics committee/institutional review board (REC/IRB) approval	_____17______
Protocol amendments	25	Plans for communicating important protocol modifications (eg, changes to eligibility criteria, outcomes, analyses) to relevant parties (eg, investigators, REC/IRBs, trial participants, trial registries, journals, regulators)	_____17______

Consent or assent	26a	Who will obtain informed consent or assent from potential trial participants or authorised surrogates, and how (see Item 32)	_____9/10____
	26b	Additional consent provisions for collection and use of participant data and biological specimens in ancillary studies, if applicable	____N/A______
Confidentiality	27	How personal information about potential and enrolled participants will be collected, shared, and maintained in order to protect confidentiality before, during, and after the trial	_____15______
Declaration of interests	28	Financial and other competing interests for principal investigators for the overall trial and each study site	_____19______
Access to data	29	Statement of who will have access to the final trial dataset, and disclosure of contractual agreements that limit such access for investigators	_____15______
Ancillary and post-trial care	30	Provisions, if any, for ancillary and post-trial care, and for compensation to those who suffer harm from trial participation	_____14______
Dissemination policy	31a	Plans for investigators and sponsor to communicate trial results to participants, healthcare professionals, the public, and other relevant groups (eg, via publication, reporting in results databases, or other data sharing arrangements), including any publication restrictions	_____17/18____
	31b	Authorship eligibility guidelines and any intended use of professional writers	_____N/A_____
	31c	Plans, if any, for granting public access to the full protocol, participant-level dataset, and statistical code	_____15______
**Appendices**			
Informed consent materials	32	Model consent form and other related documentation given to participants and authorised surrogates	____N/A_______
Biological specimens	33	Plans for collection, laboratory evaluation, and storage of biological specimens for genetic or molecular analysis in the current trial and for future use in ancillary studies, if applicable	____N/A_______

*It is strongly recommended that this checklist be read in conjunction with the SPIRIT 2013 Explanation & Elaboration for important clarification on the items. Amendments to the protocol should be tracked and dated. The SPIRIT checklist is copyrighted by the SPIRIT Group under the Creative Commons "Attribution-NonCommercial-NoDerivs 3.0 Unported" license.
